# Comparative mitogenomics of Braconidae (Insecta: Hymenoptera) and the phylogenetic utility of mitochondrial genomes with special reference to Holometabolous insects

**DOI:** 10.1186/1471-2164-11-371

**Published:** 2010-06-11

**Authors:** Shu-jun Wei, Min Shi, Michael J Sharkey, Cornelis van Achterberg, Xue-xin Chen

**Affiliations:** 1 State Key Laboratory of Rice Biology, Zhejiang University, Hangzhou 310029, China; 2 Ministry of Agriculture Key Laboratory of Molecular Biology of Crop Pathogens and Insects, Institute of Insect Sciences, Zhejiang University, Hangzhou 310029, China; 3 Institute of Plant and Environmental Protection, Beijing Academy of Agriculture and Forestry Sciences, Beijing 100097, China; 4 Department of Entomology, University of Kentucky, Lexington KY 40546-0091, USA; 5 Department of Entomology, Nationaal Natuurhistorisch Museum, Postbus 9517, 2300 RA Leiden, Netherlands

## Abstract

**Background:**

Animal mitochondrial genomes are potential models for molecular evolution and markers for phylogenetic and population studies. Previous research has shown interesting features in hymenopteran mitochondrial genomes. Here, we conducted a comparative study of mitochondrial genomes of the family Braconidae, one of the largest families of Hymenoptera, and assessed the utility of mitochondrial genomic data for phylogenetic inference at three different hierarchical levels, i.e., Braconidae, Hymenoptera, and Holometabola.

**Results:**

Seven mitochondrial genomes from seven subfamilies of Braconidae were sequenced. Three of the four sequenced A+T-rich regions are shown to be inverted. Furthermore, all species showed reversal of strand asymmetry, suggesting that inversion of the A+T-rich region might be a synapomorphy of the Braconidae. Gene rearrangement events occurred in all braconid species, but gene rearrangement rates were not taxonomically correlated. Most rearranged genes were tRNAs, except those of *Cotesia vestalis*, in which 13 protein-coding genes and 14 tRNA genes changed positions or/and directions through three kinds of gene rearrangement events. Remote inversion is posited to be the result of two independent recombination events. Evolutionary rates were lower in species of the cyclostome group than those of noncyclostomes. Phylogenetic analyses based on complete mitochondrial genomes and secondary structure of *rrnS *supported a sister-group relationship between Aphidiinae and cyclostomes. Many well accepted relationships within Hymenoptera, such as paraphyly of Symphyta and Evaniomorpha, a sister-group relationship between Orussoidea and Apocrita, and monophyly of Proctotrupomorpha, Ichneumonoidea and Aculeata were robustly confirmed. New hypotheses, such as a sister-group relationship between Evanioidea and Aculeata, were generated. Among holometabolous insects, Hymenoptera was shown to be the sister to all other orders. Mecoptera was recovered as the sister-group of Diptera. Neuropterida (Neuroptera + Megaloptera), and a sister-group relationship with (Diptera + Mecoptera) were supported across all analyses.

**Conclusions:**

Our comparative studies indicate that mitochondrial genomes are a useful phylogenetic tool at the ordinal level within Holometabola, at the superfamily within Hymenoptera and at the subfamily level within Braconidae. Variation at all of these hierarchical levels suggests that the utility of mitochondrial genomes is likely to be a valuable tool for systematics in other groups of arthropods.

## Background

Most animal mitochondrial genomes are about 16 Kb in size and contain 37 genes: 13 protein-coding genes, 22 transfer RNA genes (tRNA) and two ribosomal RNA genes (rRNA) [1]. Additionally, an A+T-rich region is present which contains essential regulatory elements for transcription and replication. It is therefore referred to as the control region [2]. Complete mitochondrial genomes provide good models for molecular evolution and abundant molecular markers for phylogenetic and population studies [3-6].

Because mitochondrial genomes are highly economized, with few intergenic regions, gene rearrangements are rare [1]. However in some lineages they are more common. For example amongst arthropods, the following groups show significant gene rearrangements: Myriapoda [7], Hymenoptera [8], hemipteroids [9-11], Acari [12,13], Araneae [14,15], and Isopoda [16,17]. These lineages therefore make ideal candidates for the study of gene rearrangement mechanisms [8,18-21].

Mitogenomic studies of the Hymenoptera have revealed many interesting features: (*i*) gene arrangements are conserved in the basal Hymenoptera, i.e., the grade Symphyta, whereas frequent gene rearrangements are observed in the derived clade, Apocrita [8,22] with approximately equal amounts of gene shuffling, inversion, and translocation [8]; (*ii*) tRNA positions are selectively neutral in all studied hymenopteran mitochondrial genomes [23]; (*iii*) various gene rearrangement mechanisms are necessary to explain the derived gene arrangement patterns in Hymenoptera [8,24], whereas amongst vertebrates, most gene rearrangement events are best explained by tandem duplication [25]; (*v*) nucleotide substitution rates are extremely high in the mitochondrial genomes of three *Nasonia *(Hymenoptera: Chalcidoidea) species, about 30 times faster than nuclear protein-coding genes [26].

The number of hymenopteran mitochondrial genomes, though high relative to other taxa, is rather limited, especially in relation to the species-richness of the order [24,26-31]. The characterization of more hymenopteran mitochondrial genomes has promise in answering evolutionary questions such as mechanisms of remote inversion events of gene rearrangement [8] and the variation of strand-specific compositional bias between Braconidae and Ichneumonidae [8,32].

Here we sequenced seven complete mitochondrial genomes of members of Braconidae, representing the subfamilies, Doryctinae, Opiinae, Microgastrinae, Cheloninae, Aphidiinae, Macrocentrinae and Euphorinae. Braconidae was selected because it is one of the largest families in Hymenoptera, second only to Ichneumonoidea, and because no complete mitochondrial genome has been reported for the family, although one of the reported mitochondrial gene rearrangement hot spots has been examined in this family [8,20]; Due to variation within the family, Braconidae is an ideal group to study the evolution of modes of parasitism [33]. Members of the subfamily Doryctinae are mostly ectoparasitic idiobionts (the host does not recover after paralysis by an ovipositing wasp and the wasp larvae feeds immediately), whereas member of the other six subfamilies are endoparasitic koinobionts (the host recovers after oviposition and develops normally for some time before it is consumed by the parasitoid). Though many efforts have been focused on the phylogeny of Braconidae, it is still a problematic group with many unresolved relationships [34-41].

Holometabolous insects represent the most successful lineages of Metazoa with 11 orders encompassing more than half of all known animal species. However, the phylogenetic relationships among orders in holometabolous insects are still controversial. Studies based on morphology or single molecular markers are limited by character quantity and quality respectively [42,43], whereas those based on complete mitochondrial genome sequences or nuclear genomes are limited by taxon sampling [44,45].

In this study, we explored some evolutionary traits of braconid mitochondrial genomes, and consequently assessed the phylogenetic utility of mitogenomics at three hierarchical levels, i.e., Braconidae, Hymenoptera and Holometabola.

## Results and Discussion

### General description Braconidae mitochondrial genomes

Two complete mitochondrial genomes from *Spathius agrili *Yang and *Cotesia vestalis *Haliday and five nearly complete mitochondrial genomes from *Aphidius gifuensis *(Ashmead), *Diachasmimorpha longicaudata *(Ashmead), *Phanerotoma flava *Ashmead, *Macrocentrus camphoraphilus *He & Chen, and *Meteorus pulchricornis *(Wesmael) were sequenced, representing seven subfamilies of Braconidae (Table 1). The regions that we failed to sequence were usually located in or around gene *nad2 *and the A+T-rich region, where extremely high A+T content, frequent gene rearrangement and stable stem-and-loop structures may have disrupted PCR and sequencing reactions. This is a common problem in sequencing of hymenopteran mitochondrial genomes [28,29,46].

**Table 1:** General information of the mitochondrial genomes from Ichneumonoidea

Species	Length (bp)	Completeness	Family	Subfamily	Accession number	Resources
*Diadegma semiclausum*	18728	Complete	Ichneumonidae	Campopleginae	EU871947	Wei et al., 2009
*Enicospilus *sp.	15300	Incomplete	Ichneumonidae	Ophioninae	FJ478177	Dowton et al., 2009
*Cotesia vestalis*	15543	Complete	Braconidae	Microgastrinae	FJ154897	This study
*Spathius agrili*	15425	Complete	Braconidae	Doryctinae	FJ387020	This study
*Phanerotoma flava*	10171	Incomplete	Braconidae	Cheloninae	GU097654	This study
*Diachasmimorpha longicaudata*	13850	Incomplete	Braconidae	Opiinae	GU097655	This study
*Macrocentrus camphoraphilus*	15801	Incomplete	Braconidae	Macrocentrinae	GU097656	This study
*Meteorus pulchricornis*	10186	Incomplete	Braconidae	Euphorinae	GU097657	This study
*Aphidius gifuensis*	11996	Incomplete	Braconidae	Aphidiinae	GU097658	This study

All genes identified in the seven mitochondrial genomes are typical animal mitochondrial genes with normal gene sizes. In all, 37 genes and an A+T-rich region were identified in the two completely sequenced mitochondrial genomes of *S. agrili *and *C. vestalis*. In the mitochondrial genome of *M. camphoraphilus*, a region spanning partial sequence of *cob *and *nad1 *was duplicated and inserted downstream of the *rrnS *- *trnM *- *trnI *- A+T-rich region and reversed. This is the first report of protein-coding gene sequence duplication in Hymenoptera mitochondrial genomes. The duplicated region led to the failure of amplification of the sequence further downstream.

All protein-coding genes start with ATN start codons and stop with TAA termination codons or truncated termination codons TA or T. Gene *nad1 *has been found to employ TTG as a start codon in some species of Hymenoptera, Coleoptera and Lepidoptera, thus minimizing intergenic spacing and avoiding overlap with adjacent genes [47-49], however, we did not discover TTG start codons in *nad1 *genes for any Braconidae.

Most transfer RNA (tRNA) genes have the usual cloverleaf structure and anticodons commonly found in insects. All *trnK *and *trnS2 *use TTT and TCT as anticodons rather than the normal CTT and GCT, respectively. The use of abnormal anticodons in these two tRNAs appears to be correlated with gene rearrangement [24].

**Figure 1:**
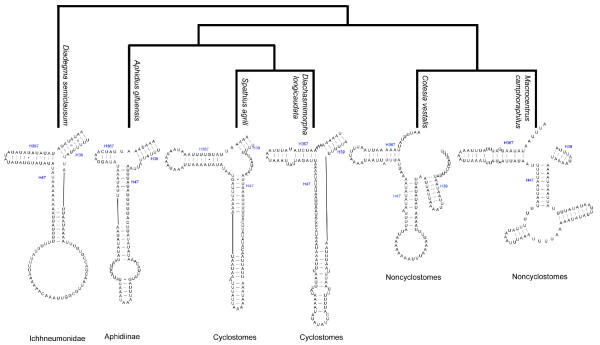
**Predicted phylogenetic relationships among five braconid species based on the secondary structures of H39, H47 and H367 in Domain I of *rrnS***. Base-pairing is indicated as follows: Watson-Crick pairs by lines, wobble GU pairs by dots and other noncanonical pairs by circles.

Two typical animal mitochondrial ribosomal RNAs (*rrnL *and *rrnS*) were sequenced in five species, i.e., *A*. *gifuensis*, *S*. *agrili*, *D. longicaudata*, *C*. *vestalis*, *M. camphoraphilus*. All of their rRNA genes conform to the secondary structure models proposed for other insects [24,50]. Compared to the secondary structure of *D. semiclausum *(Hymenoptera: Ichneumonidae) [24], these five species of Braconidae lack H9 and H17 in *rrnS *suggesting that it may be a widespread feature in Braconidae. In domain I, H37, H47 and H367 were variable among these five species. All the above three structures were present in *A. gifuensis*, *S. agrili *and *D. longicaudata*, as in *D. semiclausum*, but there was a big internal bulge at the bottom of H47 in *A. gifuensis*. In *C. vestalis *and *M. camphoraphilus*, H39 is formed by a continuous segment. The structures of H37, H47 and H367, suggest that Cyclostomes and Noncyclostomes are monophyletic and that Aphidiinae are the sister-group to the cyclostomes (Figure 1), congruent with the structures inferred from all protein coding genes (see below).

### A+T-rich region

The A+T-rich region is believed to be involved in the regulation of transcription and control of DNA replication, characterized by five elements: (1) a polyT stretch at the 5'end of the A+T-rich region, which may be involved in the control of transcription and/or replication initiation; (2) a [TA(A)]_n_-like stretch following the polyT stretch; (3) a stem and loop structure, which may be associated with the second strand-replication origin; (4) a TATA motif and a G (A)_n_T motif flanking the stem and loop structure and (5) a G+A rich sequence downstream of the stem and loop structure [2].

**Figure 2:**
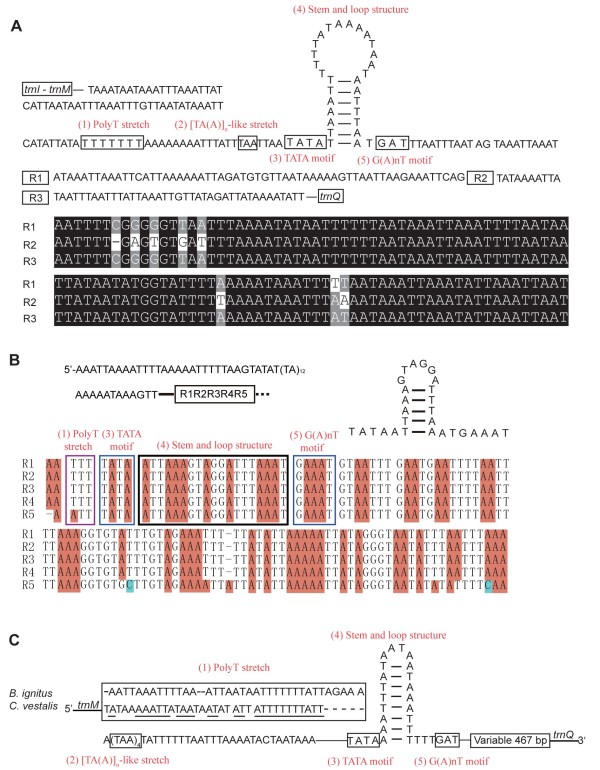
**Structural elements of A+T-rich region in three braconid mitochondrial genomes**. (A) Structure of *Spathius agrili *mitochondrial A+T-rich region. Three repeat sequences are aligned. (B) Structure of *Diachasmimorpha longicaudata *mitochondrial A+T-rich region. Five repeat sequences including three elements were aligned. (C) Structure of *Cotesia vestalis *mitochondrial A+T-rich region. PolyT stretches were compared in *C. vestalis *and *Bombus ignitus*. Short dashes indicate gaps; underlines of the PolyT stretch sequence indicate conserved region between *C. vestalis *and *B. ignitus*.

A+T-rich regions were successfully sequenced for species, *S. agrili*, *D. longicaudata*, *C. vestalis *and *M. camphoraphilus*. Elements presumed to be involved in genome replication and transcription were found in all sequenced A+T-rich regions except that of *M. camphoraphilus *(Figure 2). The A+T-rich region in the mitochondrial genome of *S. agrili *is 578 bp long, with an A+T content of 93.6%, whereas in *C. vestalis *it is 571 bp long, with an A+T content of 92.6%. In the A+T-rich region of *S. agrili*, three repeat sequences are present downstream of the identified elements. In the A+T-rich region of *D. longicaudata*, identified elements are included in five repeat elements. However, all identified elements in the A+T-rich region of these three braconid species were found to be located in opposite directions and strands relative to those of other insects [2,30], indicating an inversion of the A+T-rich region in these species [16]. This is the first time that an inversion of the A+T-rich region has been demonstrated structurally for insects.

### Gene rearrangement

**Figure 3:**
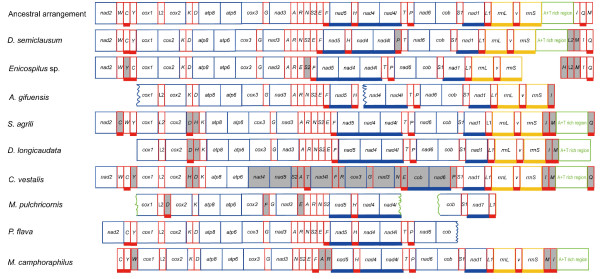
**Gene arrangement of seven braconid mitochondrial genomes sequenced in this study**. Abbreviations for the genes are as follows: *cox1*, *cox2*, and *cox3 *refer to the cytochrome oxidase subunits, *cob *refers to cytochrome b, and *nad1*-nad6 refer to NADH dehydrogenase components, *rrnL *and *rrnS *refer to ribosomal RNAs. Transfer RNA genes are denoted by one letter symbols according to the IPUC-IUB one-letter amino acid codes. *L1*, *L2*, *S1*, *S2 *denote *tRNA^Leu(CUN)^*, *tRNA^Leu(UUR)^*, *tRNA^Ser(AGY)^*, *tRNA^Ser(UCN)^*, respectively. Boxes with underscores indicate that the gene is encoded in minority strand. Shaded boxes indicate that the gene was rearranged compared with ancestral arrangement of Hexapoda.

Gene rearrangement events occurred in all seven species of Braconidae (Figure 3). All rearranged genes were tRNA, except for *C. vestalis*, in which seven of the 13 protein-coding genes and 14 tRNA genes changed their positions or/and directions. Hymenoptera have been shown to have an accelerated rate of mitochondrial gene rearrangement [8,21], however, protein-coding gene rearrangement has only been found in the mitochondrial genomes of three *Nasonia *(Chalcidoidea:Pteromalidae) species, in which at least a large segment including six protein-coding genes and three tRNAs are inverted [26]. Gene rearrangement events in *C. vestalis *and the three *Nasonia *species indicate that large-scale protein-coding gene rearrangement are probably independent events in Hymenoptera.

We compared gene rearrangement rates among braconid mitochondrial genomes in the region from *cox1 *to *trnH*, which was successfully sequenced in all seven species; gene rearrangement rates were unequal among them. Gene arrangement pattern was conserved in *A. gifuensis *and *P. flava*, and derived in the other five species. Of the two microgastroid species *P. flava *and *C. vestalis *[20,41,51], the former is conserved while the latter was markedly rearranged in gene pattern. In conclusion, mitochondrial gene rearrangement rate was not taxonomically correlated at our level of investigation of the Braconidae.

Gene rearrangement events comprise three classes, i.e., translocation, local inversion (inverted but remaining in the position), and shuffling with remote inversion (translocated and inverted) [21]. All determined *trnI *and *trnM *were inverted and translocated, forming the arrangement pattern *trnI*(-) - *trnM*(-) - A+T-rich region - *trnQ *in *S. agrili*. *D. longicaudata *and *C. vestalis*, and *trnM*(-) - *trnI*(-) - A+T-rich region - *trnQ *in *M. camphoraphilus*.

In the three species *S. agrili*, *D. longicaudata *and *C. vestalis*, *trnH *was inverted and translocated (remote inversion) to the junction of *cox2 *and *atp8*. The arrangement pattern, *trnD*(-) - *trnH *- *trnK *or *trnH*(-) - *trnD *- *trnK*, has been found in many subfamilies of Braconidae. It has been reported that both the inversion of *trnD *and the remote inversion of *trnH *are independent evolutionary events in this family [8,20]. In the two reported species of Doryctinae, *Jarra phorocantha *and *Heterospilus *sp., the ancestral arrangement pattern of *trnK *- *trnD occurs *[8], which suggests that the gene rearrangement event in *S. agrili *occurred after the origin of the subfamily.

### Gene rearrangement mechanism

Mechanisms for the three classes of gene rearrangement events in hymenopteran mitochondrial genomes are widely discussed [8,21]. Inter/intro mitochondrial genome combination is presumed to be the most plausible explanation for local inversions. *trnY *in *C. vestalis *is the only locally inverted gene in these seven braconid species, except for the region from *trnE *to *cob *in *C. vestalis*. The duplication/random loss model, and the intramitochondrial genome recombination and duplication/nonrandom loss model are possible mechanisms to explain translocation. Of these, intramitochondrial genome recombination is presumed to be more common. Shuffling is thought to be the result of duplication/random loss. The tRNA clusters *trnW *- *trnC *- *trnY *and *trnK *- *trnD *are frequently shuffled regions. *trnD *was not only shuffled but also inverted. This may have been the result of two independent events caused by separate mechanisms.

**Figure 4:**
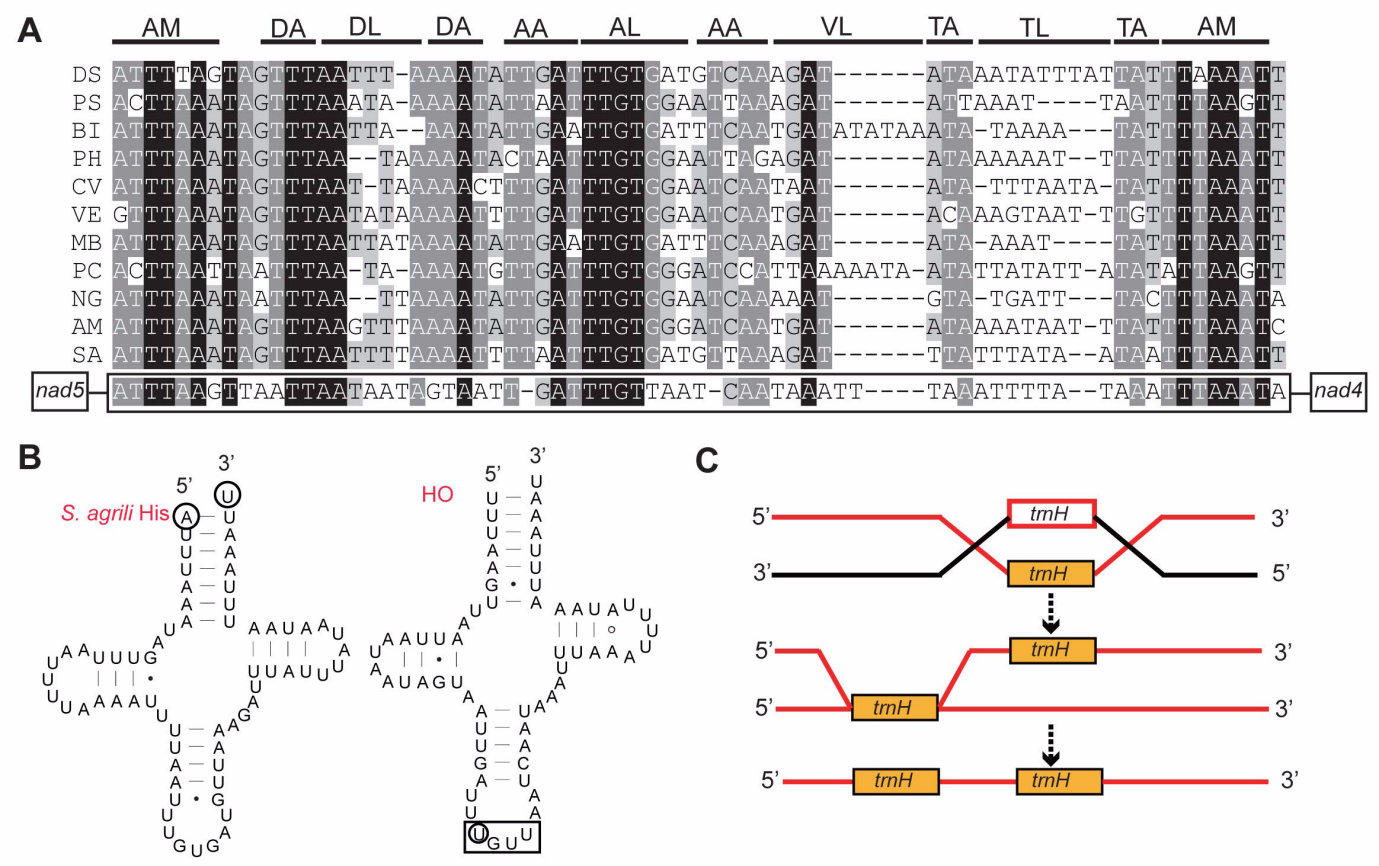
**Mechanism of *trnH *remote inversion in *Spathius agrili *mitochondrial genome**. (A) Presumed pseudo-*trnH *sequence (HO) and 10 hymenopteran *trnH *sequences are aligned according to their secondary structures. AM: Accepter arm, DA: D-loop arm, DL: D-loop, AA: Anticodon arm, AL: Anticodon loop, VL: Variable loop, TA: T Ψ C arm, TL: T Ψ C loop. (B) Secondary structure *trnH *is predicted in tRNAscan-SE search server [82] and HO is predicted manually. The inserted uracil in the anticodon is showed by a circle. (C) Recombination of two strands with opposite orientations leads the inversion of *trnH*, and the following recombination events lead the duplication of trnH. DS: *Diadegma semiclausum*, PS: *Primeuchroeus *spp., BI: *Bombus ignites*; PH: *Polistes humilis*, CV: *Cotesia vestalis*, VE: *Vanhornia eucnemidarum*, MB: *Melipona bicolor*, PC: *Perga condei*, NG: *Nasonia giraulti*, AM: *Apis mellifera*, SA: *Spathius agrili*.

Remote inversion is a common rearrangement event in braconid mitochondrial genomes (*trnH*, *trnI *and *trnM*). However, remote inversion could not be deduced in previous studies due to incompleteness of sequence data [8,21]. In *S. agrili*, an inverted pseudo-*trnH *sequence is located between *nad4 *and *nad5*, the ancestral position of *trnH *(Figure 4A,B). Pseudo-genes are usually considered to be genomic evidence for the duplication and loss model of rearrangement [52-54]. The presence of the pseudo-*trnH *sequence in the same position and opposite direction to the ancestral *trnH *indicates that *trnH *was inverted before translocation. Both the inversion and translocation events may be the result of recombination as detailed above. During the inversion process, the two recombined sequences occur in opposite directions, whereas during translocation, the directions of the two recombined sequences are the same (Figure 4C). In *C. vestalis*, a 23 bp and in *D. longicaudata *a 52 bp intergenic region were found in the ancestral location of *trnH*, which might indicate a similar rearrangement process as *trnH *in *S. agrili*. The pseudo-*trnH *sequence in *S. agrili *is well conserved, whereas the ancestral sequence of *trnH *in *C. vestalis *and *D. longicaudata *were eliminated sometime after the loss of gene function [55]. Our results indicate that remote inversion may be caused by two separate recombination events.

Since *trnI*, *trnM *and the A+T-rich region were all inverted, separated remote inversions would make the rearrangement in this region extremely complicated. Therefore, it is more likely that *trnI *and *trnM *were inverted simultaneously. Thus, before the inversion of this region, *trnQ *would have been shuffled to form an intermediate pattern of A+T-rich region - *trnM *- *trnI - trnQ*. We observed a *trnM *- *trnI - trnQ *arrangement pattern in the mitochondrial genome of *Diadegma semiclausum*, an Ichneumonidae, the sister-group of Braconidae [24]. This suggests that *trnM *- *trnI - trnQ *is ancestral, and *trnM *- *trnI *- A+T-rich region is a derived pattern in Braconidae. Recombination is more likely to explain the inversion of A+T-rich region - *trnM *- *trnI - trnQ *based on the parsimony criterion.

**Figure 5:**
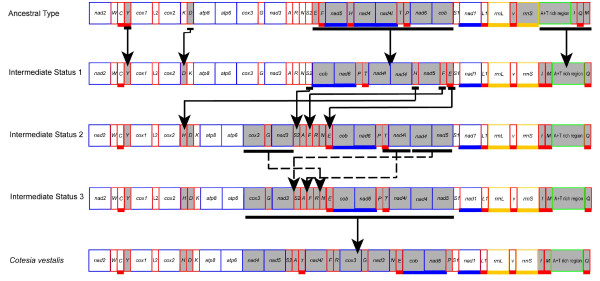
**Putative gene rearrangement events in *Cotesia vestalis *mitochondrial genome**. Four regions underwent gene rearrangements. Rearranged genes are shown in gray. The region from *cox3 *to *cob *experienced protein-coding gene rearrangements, and three types of rearrangement events might happen: large-scale inversion, tRNA rearrangement and small region rearrangement. The intermediate statuses are used to show different types of gene rearrangement events, but not the rearrangement process.

In *C. vestalis*, it seems that all gene rearrangement events at *atp6 *- *trnS1 *junction are difficult to trace; however the parsimony criterion implies that three types of rearrangement events may have occurred (Figure 5): (1) Early large-scale inversion. Genes from *trnE *to *cob *were inverted as a whole before changing their relative positions. A large-scale inversion is present in the three sequenced *Nasonia *species, where either region *nad3 *to *cox1 *or *trnF *to *cob *is inverted. If an inverted region from *trnE *to *cob *is present in Ichneumonoidea, it may be a case of convergent evolution between the mitochondrial genomes of Chalcidoidea and Ichneumonoidea, because gene arrangement of this region is conserved in *D. semiclausum *(Ichneumonoidea) [24]. Since inversion cannot be explained by replication-slippage-based models [19], recombination is the most likely mechanism for this rearrangement event.

(2) tRNA translocations. The six tRNA genes between *nad3 *and *nad5 *in their ancestral position are heavily rearranged. And the most apparently conserved order of this tRNA cluster is *trnA *- *trnR *- *trnN*, which indicates that the derived relative positions of these tRNA genes are most likely the result of translocations of *trnF *and *trnE *and shuffling of *trnS2*. Nevertheless, *trnH *may have been inverted and translocated to the junction of *cox2 *and *atp8 *before the large-scale inversion event. The most possible mechanisms of gene translocation and shuffling are recombination, rather than tandem duplication followed by deletion (TDRL), which might change the remnant order of tRNA and the neighbouring protein-coding genes.

(3) Small-scale translocations. Four gene boundaries, i.e., *nad4 *- *nad5*, *trnT *- *nad4l*, *cox3 *- *trnG *- *nad3 *and *cob *- *nad6 *- *trnP*, were translocated after the large-scale inversion at the junction of *atp6 *- *trnS1*. Although tRNA gene rearrangement is more frequent than that of protein-coding genes, translocation of three gene boundaries is more parsimonious than tRNA gene rearrangement. Recombination is also favoured in these small-scale translocation events, because neither pseudo-genes nor large intergenic spacers are present in the boundaries of these rearranged genes, which are the intermediate state of the TDRL model [52].

### Nucleotide composition

Braconidae mitochondrial genomes have high A+T content, a characteristic typical of other hymenopterans, with values from 82.4% to 87.2%.

Strand asymmetry (strand compositional bias) are usually reflected by AT skew, as expressed by (A-T)/(A+T), and GC skew, as expressed by (G-C)/(G+C) [56]. Strands of insect mitochondrial genome are discriminated as majority strand (encoding most genes) and minority strand (the other strand) [57]. In all braconid mitochondrial genomes, the signs of GC skew on the entire majority strand and all protein-coding genes were reversed relative to those of Ichneumonidae (Table 2).

**Table 2:** Nucleotide composition of Ichneumonoidea mitochondrial genomes

Species	Whole genome sequences				All protein-coding genes		
	AT skew	GC skew	A+T%		AT skew	GC skew	A+T%
*Diadegma semiclausum*	0.01	-0.20	87.40		-0.12	-0.03	84.75
*Enicospilus *sp.	-0.02	-0.18	85.10		-0.11	-0.02	85.12
*Aphidius gifuensis*	-0.06	0.05	84.70		-0.16	0.09	84.80
*Spathius agrili*	-0.07	0.19	84.00		-0.15	0.16	83.25
*Diachasmimorpha longicaudata*	-0.09	0.22	82.40		-0.16	0.11	81.52
*Cotesia vestalis*	-0.09	0.10	87.20		-0.16	0.06	86.81
*Phanerotoma flava*	-0.07	0.28	84.50		-0.16	0.19	85.38
*Macrocentrus camphoraphilus*	-0.05	0.09	86.60		-0.13	0.10	86.47
*Meteorus pulchricornis*	-0.06	0.14	83.30		-0.16	0.11	83.10

Hassanin et al. (2005) suggested that strand asymmetry is best reflected in the GC skew. Hence, all braconid species in the present study show reversal of strand asymmetry, as in some other arthropods [16,58-61], flatworms [62], brachiopods [63], echinoderms [64] and fish [65].

Inversion of replication origin located in the A+T-rich region would lead to reversal of strand asymmetry [16,59,66], which were proved by examination of regulatory elements in A+T rich region in three sequenced braconid mitochondrial genomes. Although further sampling is necessary the present evidence suggests that reversal of strand asymmetry and inversion of the A+T-rich region is a synapomorphy for members of Braconidae.

### Evolutionary rate

**Figure 6:**
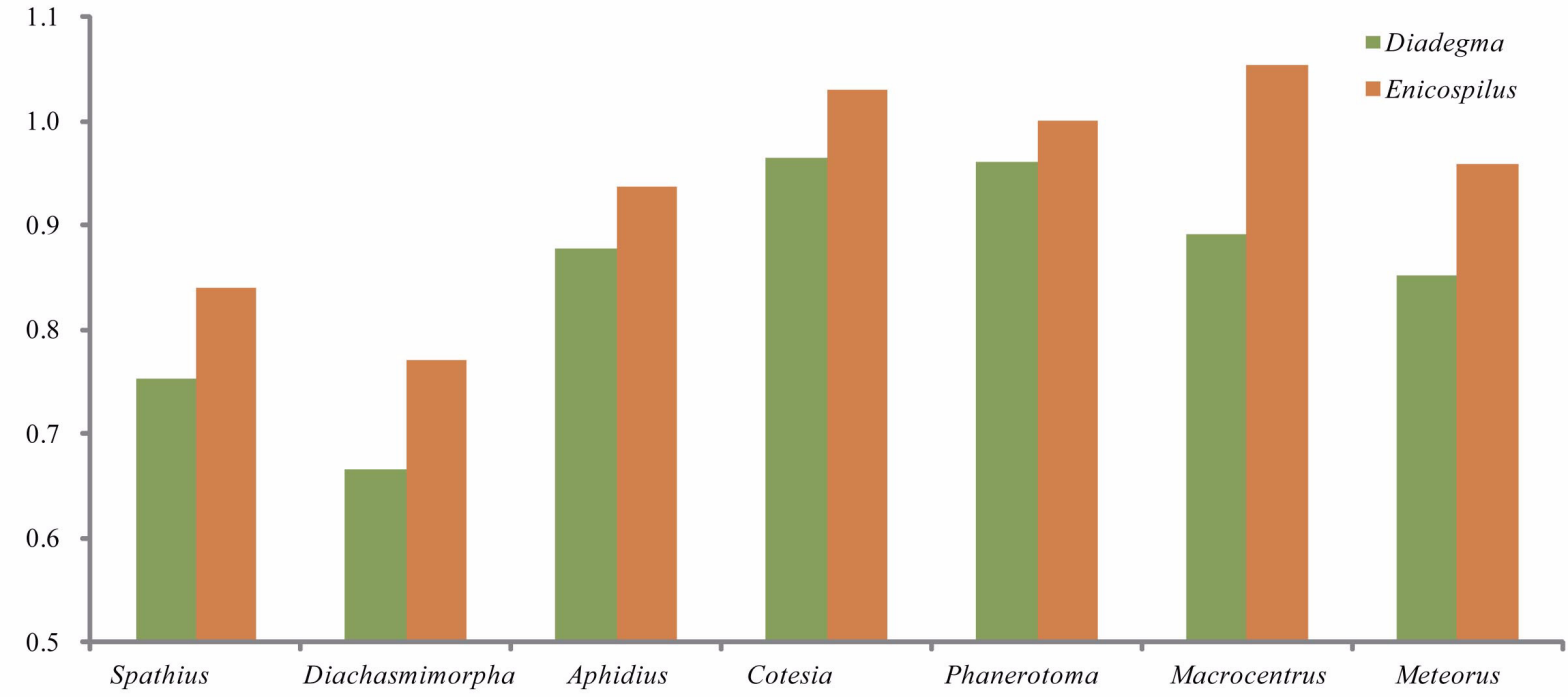
**Evolutionary rates of braconid mitochondrial genomes**. The ration of the number of nonsynonymous substitutions per nonsynonymous site (Ka) and the number of synonymous substitutions per synonymous site (Ks) for each braconid mitochondrial genomes, using that of *Diadegma semiclausum *or *Enicospilus *sp. as reference sequences.

The rate of non-synonymous substitutions (Ka), the rate of synonymous substitutions (Ks) and the ratio of the rate of non-synonymous substitutions to the rate of synonymous substitutions (Ka/Ks) were calculated for each braconid mitochondrial genome using *D. semiclausum *or *Enicospilus *sp. (Hymenoptera: Ichneumonidae) as reference sequences (Figure 6). Species of non-cyclostomes showed higher evolutionary rates than those of cyclostomes. *C. vestalis*, *P. flava *and *M. camphoraphilus *showed the highest Ka/Ks ratios, indicating that the mitochondrial genomes with high gene rearrangement rates have high evolutionary rates, but those with low gene rearrangement rate are not constrained in their evolutionary rate.

### Phylogenomics of Braconidae

**Figure 7:**
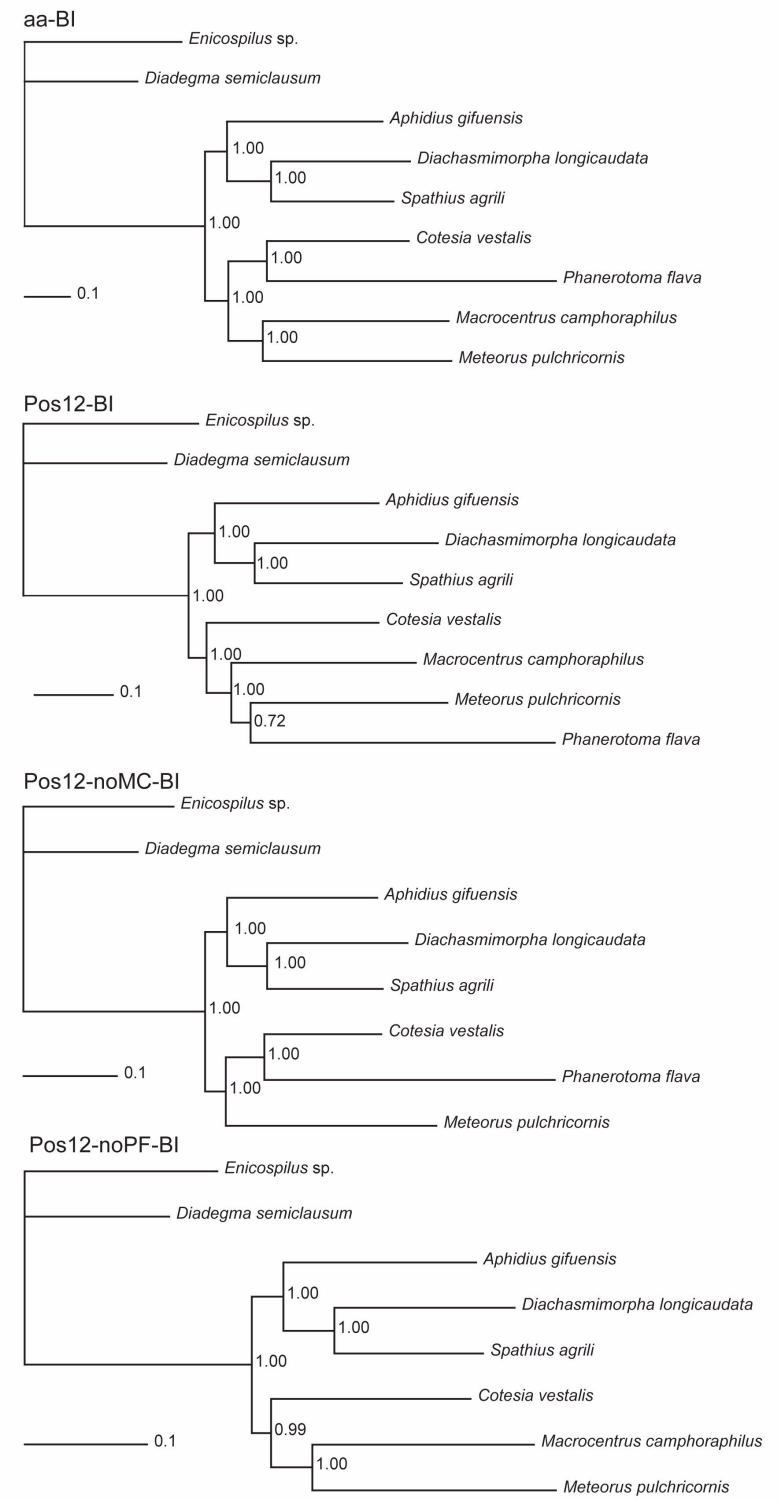
**Braconidae phylogeny based on complete mitochondrial genome sequences**. Bayes phylogenetic trees for all seven braconid species based on amino acid sequence (aa) and nucleotide sequences of first and second codon positions (Pos12-BI) of all protein-coding genes except *nad2*, for species without *Macrocentrus camphoraphilus *(Pos12-noMC-BI) or *Phanerotoma flava *(Pos12-noPF-BI) were present. Bootstrap support values followed by Bayesian posterior probabilities (BPP) are shown at the right of respective nodes.

A sister-group relationship between *S. agrili *(subfamily Doryctinae) and *D. longicaudata *(subfamily Opiinae) were recovered in all analyses employing different inference methods and data. This is consistent with the widely accepted group, the cyclostomes. *A. gifuensis *(subfamily Aphidiinae) are firmly placed as the sister-group to the cyclostomes. Four species *C. vestalis *(Microgastrinae), *P. flava *(Cheloninae), *M. camphoraphilus *(Macrocentrinae) and *M. pulchricornis *(Euphorinae) constitute the other major group, the noncyclostomes. However, the internal relationships among noncyclostome subfamilies were not well resolved. In previous studies, Microgastrinae and Cheloninae were generally recovered in a clade referred to as microgastroids, while Macrocentrinae and Euphorinae form part of the clade, helconoids [20]. The latter group was recovered in our study in our Bayes analyses based on all amino acid sequences and it was also recovered in most likelihood analyses based on the first and second codon positions of protein-coding genes. In other analysis, Cheloninae was recovered as the sister-group of Macrocentrinae or Euphorinae, with Microgastrinae sister to the remaining noncyclostomes. Support values for the relationships Cheloninae + Euphorinae or Cheloninae + Macrocentrinae were higher than Microgastrinae + Cheloninae (well corroborated in many other analyses) and Macrocentrinae + Euphorinae. When *M. camphoraphilus *or *P. flava *were excluded from the Bayes analyses, both microgastroids and helconoids were recovered with improved support values (Figure 7, Additional file 1).

Subfamilies of braconid have traditionally been divided into two groups, cyclostomes and noncyclostomes [20,35,39]. These clades were firmly resolved in all our analyses of seven representative subfamilies. Aphidiinae was also recovered as sister-group to cyclostomes by Dowton (2002) and Zaldivar-Riverón (2006). Our results obviously support the sister group relationship between Aphidiinae and cyclostomes. Sampling of only seven of the 40 subfamilies of Braconidae may be the cause of the misplacement of Cheloninae in some analyses. Our analyses indicate that mitochondrial genome sequence data has the potential to resolve the phylogenetic relationships among braconid subfamilies with increased taxon sampling.

### Phylogenomics of Holometabola with an emphasis on Hymenoptera

**Figure 8:**
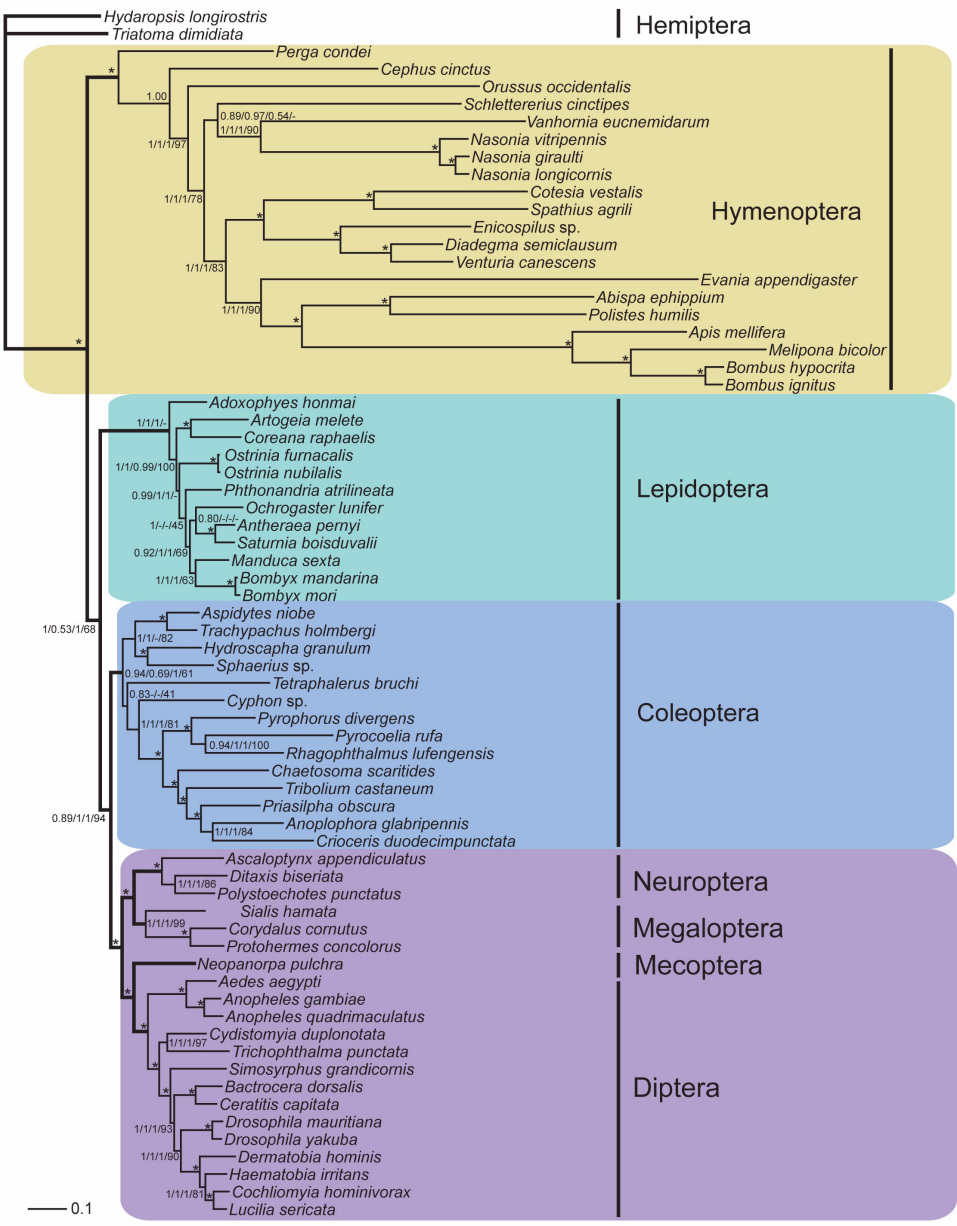
**A combined holometabolous phylogenetic tree based on all mitochondrial protein-coding genes**. Bootstrap support values for the nodes inferred from four analyses (by Bayes inference method based on first, second and RY-coded third codon positions, first and second codon positions, and amino acid sequences, and by most likelihood method based on first, second and RY-coded third codon positions of all mitochondrial protein-coding genes) were shown sequentially separated by "/". "*" indicates that the node were fully supported by all four inferences; "-" indicates that the node was not recovered by the corresponding inference.

We performed 12 phylogenetic analyses using combinations of four datasets and three analytical methods to test the utility of mitochondrial genome sequences among hymenopteran superfamilies and holometabolus orders (Additional file 2). The third codon position has proved to be less restricted by purifying selection [67] and easily saturated with substitutions, thus it is usually ignored in phylogenetic analyses [68], and, Cameron et al. (2007b) found it to be a major source of homoplasy. In our analyses, the exclusion of the third codon improved the topology of the tree, furthermore, the recoding of purines and pyrimidines into R and Y showed improvement of both topology and support values (Figure 8). Baysian (BI) trees tend to reflect accepted topologies better than maximum likelihood (ML) and maximum parsimony (MP) trees. In the MP trees, many nodes were unresolved, and in ML, many nodes had low support values.

Monophyly of the seven in-group orders was recovered in most analysis. Hymenoptera was recovered at the base of all homometabolous lineages, except in most likelihood analyses based on amino acid and protein-coding positions, in which Hymenoptera and Lepidoptera were recovered as sister-groups with low support value. In this study, we included mitochondrial genome sequences from Neuroptera and Mecoptera for the first time. Mecoptera was recovered as a sister-group of Diptera. Neuropterida (Neuroptera + Megaloptera in this study) is supported across all analyses, forming the sister-group to (Diptera + Mecoptera), differing from the presently preferred sister-group relationship of Neuropterida and Coleoptera [43].

In the previous analysis using complete mitochondrial genome sequence data, the relationships among holometabolous orders were not well resolved [45,68]. Here, increased taxon sampling generated more stable phylogenetic relationships. The basal position of Hymenoptera is congruent with the analysis based on whole nuclear genome sequences [44].

Within Hymenoptera, three species of Symphyta representing three superfamilies and three families and 17 species of Apocrita representing seven superfamilies and eight families were used in analysis. Symphyta was shown to be paraphyletic; Apocrita was monophyletic with Orussoidea as its sister-group. Two major clades were recovered within Apocrita: Stephanoidea + Proctotrupomorpha, and Ichneumonoidea + (Evanioidea + Aculeata). Proctotrupomorpha (Proctotrupoidea + Chalcidoidea in this study), Ichneumonoidea (Ichneumonidae + Braconidae) and Aculeata ((Eumeninae + Vespinae) + Apoidea) in this study) were strongly supported in all analysis. Evaniomorpha proposed in [69] was not recovered: Evanoidea was recovered as sister-group to Aculeata, while Stephanoidea was sister to Proctotrupomorpha. Ichneumonoidea was the sister group of (Evanoidea + Aculeata). All nodes among Hymenoptera were perfectly supported in BI analyses except that of Stephanoidea + Proctotrupomorpha. In the topology based on amino acid sequences using BI, Stephanoidea was recovered as the sister-group of (Ichneumonoidea + (Evanoidea + Aculeata)), with a low support value of 0.54. In all analyses the ancestral position of *Nasonia vitripennis *among three *Nasonia *species is supported, which is congruent with the analyses based on nuclear gene sequence data and phylogeny of *Wolbachia *bacteria that they host [26,70].

Phylogenetic relationships among Hymenoptera at the superfamily level remain controversial [71]. Many well accepted phylogenetic relationships were recovered, such as the sister group relationship between Orussoidea and Apocrita, Apocrita, Proctotrupomorpha, Aculeata (or Vespomorpha) [69,72-78]. Evaniomorpha was frequently recovered as polyphyletic [71,75,76,79]. Castro and Dowton (2006) recovered a similar relationship among Ichneumonoidea, Evaniidae and Aculeata. The difference is that a group including Stephanidae was sister to Aculeata.

Within Diptera, well established relationships were recovered in nearly all of the analyses. Suborder relationships within Coleoptera were less stable. Adephaga and Myxophaga were recovered as sister group and then sister to Polyphaga. However, Archostemata was either recovered as sister-group to Polyphaga, or to Adephaga. Monophyly of the suborder Polyphaga is recovered in most analyses with internal relationships of *Cyphon *+ (Elateroidea + (*Chaetosoma *+ (*Tribolium*+ (*Priasilpha *+ (*Anoplophora *+ *Crioceris*))))). The intraordinal relationships of Lepidoptera are also unstable among datasets and methods. BI analyses supported early divergent of Rhopalocera (Lycaenidae + Pieridae in this study) most ML and MP analyses supported that of Tortricidae (Tortricoidea). Monophyly of Bombycoidea (Saturniidae + (Sphingidae + Bombycidae) in this study) was firmly supported in most analysis. The position of Lepidoptera and its internal relationships is likely negatively affected that the small sample of all taxa all of which were restricted to the clade Apoditrysia. We will not discuss the relationships among Coleoptera and Lepidoptera further since phylogenetic relationships are not robustly supported. However, our results might be of service to future researchers.

## Conclusions

In this study, we reported seven complete or nearly complete mitochondrial genomes representing seven subfamilies of Braconidae. Four sequenced A+T-rich regions were shown to be inverted, as reported for species of Philopteridae (Phthiraptera) and Aleyrodidae (Hemiptera). Reversal of strand asymmetry was found in all seven sequenced mitochondrial genomes, which is correlated with the inversion of the A+T-rich region, indicating that inversion of A+T-rich region might be a ground-plan feature of braconid mitochondrial genomes. Mitochondrial gene rearrangement rates differed markedly among Braconidae, revealing that gene rearrangement might be more diverse than that previously reported [8,21,22,29]. Among different rearrangement events, remote inversion was common in Braconidae.

Noncyclostome species have a higher evolutionary rate in mitochondrial genome sequences than those of cyclostome species. Those species with high gene rearrangement rates also have high nucleotide sequence evolutionary rates.

Phylogenetic analysis using complete mitochondrial genomes sequences recovered most of the well corroborated phylogenetic relationships among major lineages of Braconidae, depending on the method and data matrix used. Cyclostomes and noncyclostomes were recovered in all analysis, and Aphidiinae was firmly recovered as be a sister-group to the cyclostomes.

Within Hymenoptera, many well accepted relationships, such as the paraphyly of Symphyta and Evaniomorpha, the sister-group relationship between Orussoidea and Apocrita, and the taxa Proctotrupomorpha, Ichneumonoidea and Aculeata, were recovered with high support values. New views among major groups in Hymenoptera are suggested, such as the sister-group relationship between Evaniidae and Aculeata. Within Diptera, relationships were very stable across analysis, but not stable among suborders of Coleoptera and major lineages of Lepidoptera.

Relationships among holometabolous orders were improved with the increase of sampling based on complete mitochondrial genome sequences and the recently suggested basal position of Hymenoptera was supported in most analysis.

In conclusion, complete mitochondrial genome data have obvious potential to infer phylogenetic relationships at the subfamily, family, ordinal levels.

## Methods

### DNA extraction, PCR amplification and sequencing

For each species, one male adult or a leg was homogenized in liquid nitrogen and total genomic DNA was extracted using the DNeasy tissue kit (Qiagen, Hilden, Germany) following manufacturer protocols.

A range of universal insect mitochondrial primers and hymenopteran mitochondrial primers modified from universal insect mitochondrial primers were used [40,57,80,81]. When necessary, species-specific primers were designed based on sequenced fragments and combined in various ways to bridge gaps. PCR and sequencing reactions were conducted following Wei at al. (2009).

### Genome annotation

Protein-coding and rRNA genes were initially identified using BLAST searches in GenBank and subsequently by alignment with genes of other insects. Protein-coding genes were translated using invertebrate mitochondrial genetic code. The tRNA searches were carried out with tRNAscan-SE search server [82]. The parameters for the tRNA scan were set for Mito/Chloromast as the source, and the Invertebrate Mito genetic code was used. When long tracts of non-coding sequence were apparent and tRNA genes were not detected using default settings, the cove cutoff score was reduced and the search repeated. Finally, those tRNA genes that could not be identified by tRNAscan-SE were inspected by eye. rRNA structures were constructed by comparison with those in other insects and algorithm-based methods as in [24,49]. All secondary structures were drawn in XRNA (developed by B. Weiser and available at http://rna.ucsc.edu/rnacenter/xrna/xrna.html).

### Evolutionary rates

The software packages DnaSP 4.0 (Rozas et al. 2003) was used to compute the number of synonymous substitutions per synonymous site (Ks) and the number of nonsynonymous substitutions per nonsynonymous site (Ka) for each braconid mitochondrial genomes, using that of *D. semiclausum *or *Enicospilus *sp. as reference sequences.

### Phylogenetic inference

For construction of the phylogenetic relationships among Braconidae, seven species with mitochondrial genomes sequenced in this study were used, representing seven subfamilies of Braconidae. *D. semiclausum *and *Enicospilus *sp., both Ichneumonidae, the family commonly accepted as the sister-group of the Braconidae, were employed as outgroups.

For construction of phylogenetic relationships among holometabolous insects, 69 species with complete or nearly complete mitochondrial genome sequences were used (Additional file 2). Twenty species were chosen from Hymenoptera, representing 10 superfamilies and 11 families [71]. Among Braconidae, *S. agrili *representing the cyclostomes and *C. vestalis *representing the noncyclostomes were used. Twelve species in nine families of the Lepidoptera, 14 species in 14 families of Coleoptera and 14 species in nine families of Diptera were included. Additionally, three species of Neuroptera, three species of Megaloptera and one species of Mecoptera were used to improve the sampling of orders among Holometabola. *Hydaropsis longirostris *and *Triatoma dimidiata *(Hemiptera) were used as outgroup taxa.

Amino acid sequences of protein-coding genes were aligned independently using ClustalX version 2.0.7 [83] with default parameters. Alignment of protein-coding genes was inferred from amino acid alignment using RevTrans [84]. Regions especially in the boundaries of genes that were aligned ambiguously were excluded in MacClade ver4.06 [85]. All protein-coding genes were concatenated following their ancestral order in insect mitochondrial genomes. Data were partitioned based on first, second and third codon positions.

Four datasets were employed in the phylogenetic analyses: amino acid sequence (aa), nucleotide sequences of first and second codon positions (Pos12), all codon positions (Pos123), first and second codon positions, and RY-coded (purines coded by R and pyrimidines coded by Y) third codon position (Pos12RY3) of all protein-coding genes. Gene *nad2 *was excluded in construction of the phylogenetic relationships of braconid subfamilies, because this gene failed to amplify in four of the seven braconid species.

Phylogenetic analyses were performed using Maximum Parsimony (MP) with PAUP* 4.0b10 [86], Maximum Likelihood (ML) with PhyML [87,88], and Bayesian Inference (BI) with MrBayes v3.1.2 [89]. The MP analyses were run with default heuristic search options except that 100 replicates of random stepwise additions were used. Bootstrap proportions (BPs) were obtained after 1000 replicates by using 10 replicates of random stepwise additions of taxa. Models of DNA substitution were estimated in Modeltest 3.7 [90]. For ML, we used a GTR+I+G model for nucleotide sequences and MtArt model for amino acid sequences with all parameters estimated. For BI, we used GTR+I+G model for four-state nucleotide sequences and a two-state substitution model with parameter I+G for RY-coded third codon position. All Bayesian analyses were conducted with four independent Markov chains run for 1,000,000 to 5,000,000 metropolis-coupled MCMC generations, with tree sampling every 100 to 500 generations and a burn-in of 2500 trees.

## Authors' contributions

Conceived and designed the experiments: SJW and MS. Performed the experiments: SJW. Analyzed the data: SJW and MS. Wrote the paper: SJW. Intellectual contributions during the design and implementation of this study, and during the writing of the manuscript: XXC, MJS and CVA. Provided funding in support of this study: XXC and MJS. All authors read and approved the final manuscript.

## Supplementary Material

Additional file 1 Phylogenetic trees constructed in this study Click here for file

Additional file 2 Species used in phylogenetic reconstruction of Holometabola Click here for file
