# Supplementary figures and images for: Comparative mitogenomics of Braconidae (Insecta: Hymenoptera) and the phylogenetic utility of mitochondrial genomes with special reference to Holometabolous insects
Source: BMC Genomics. 2010 Jun 11;11:371. doi: 10.1186/1471-2164-11-371 (PMC2890569; doi:10.1186/1471-2164-11-371)

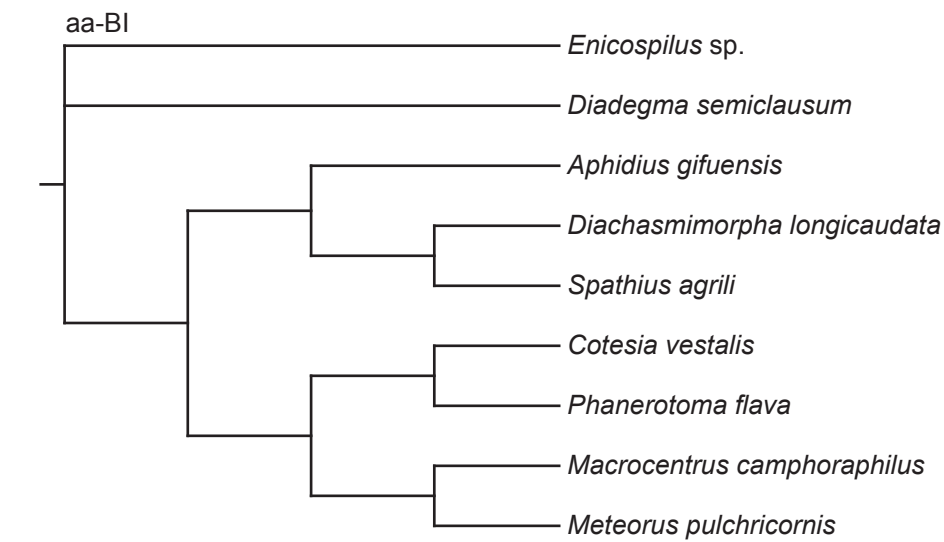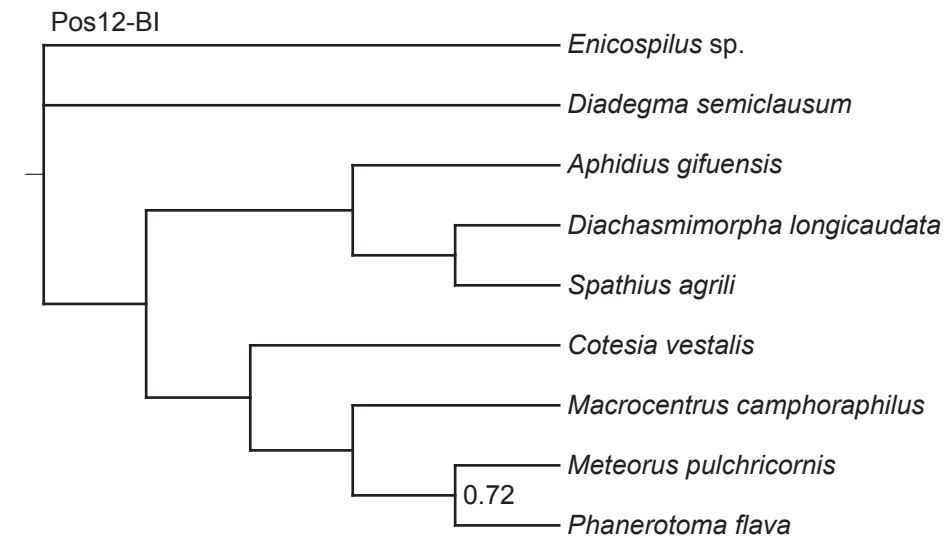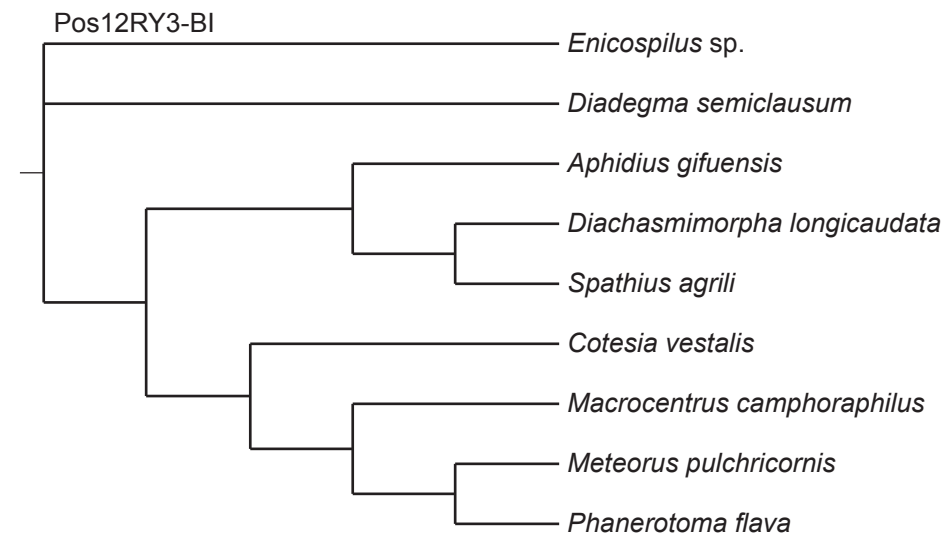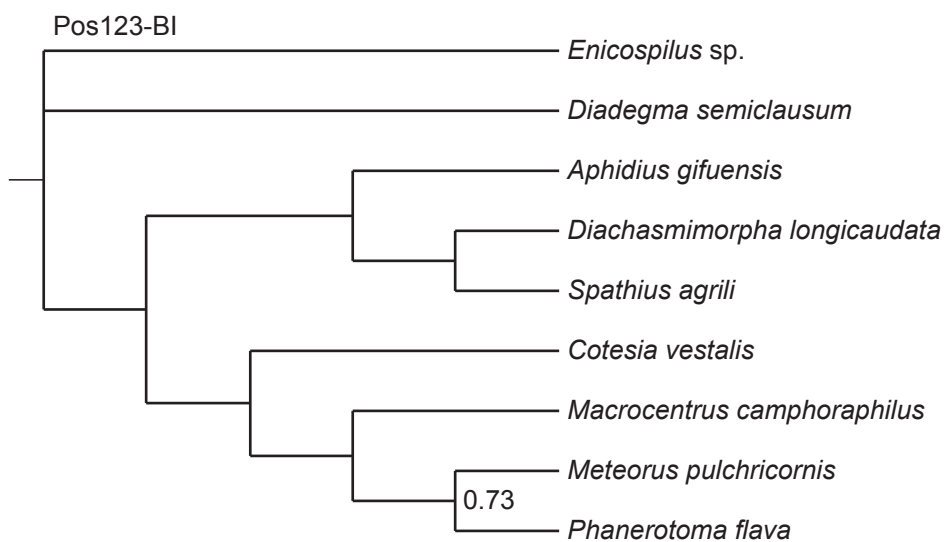

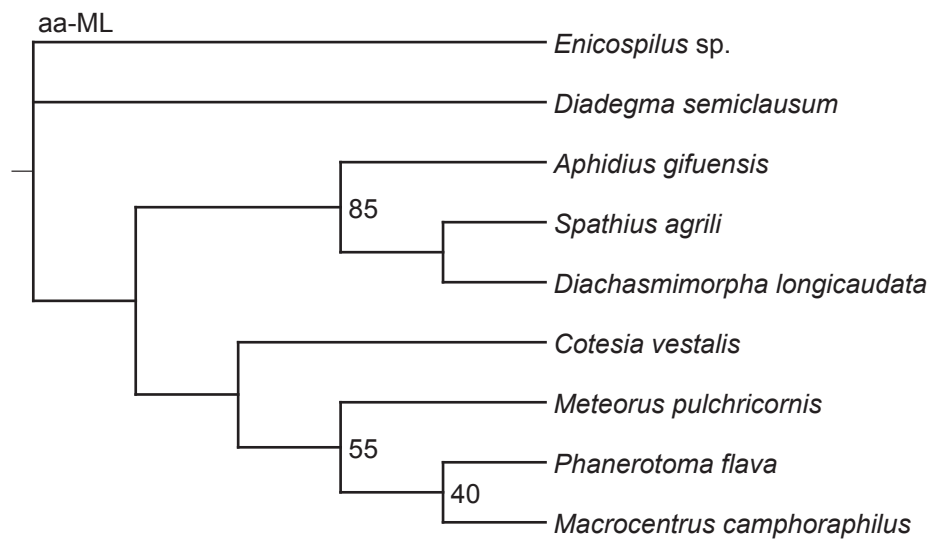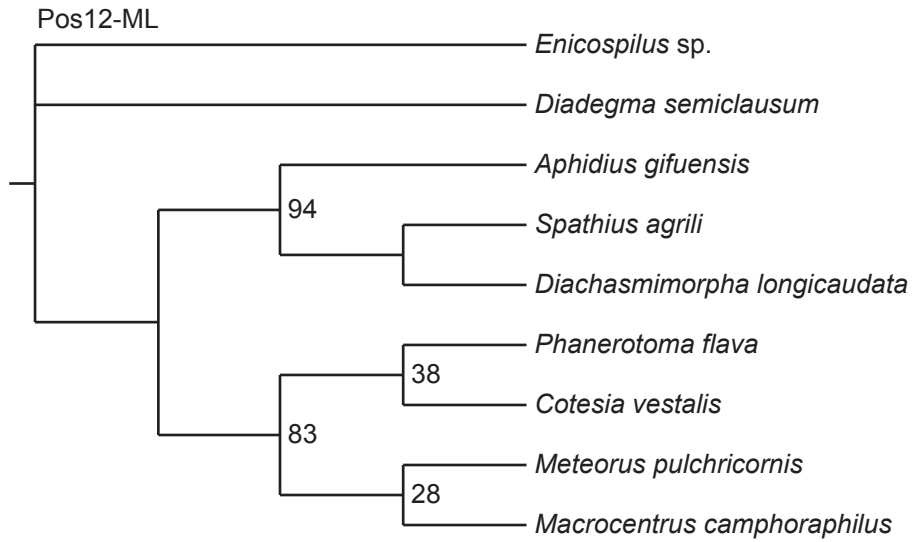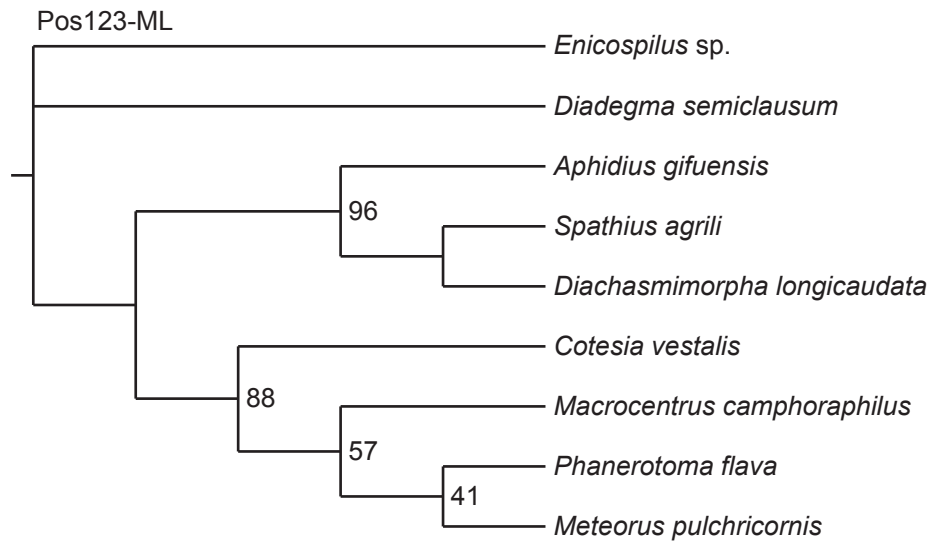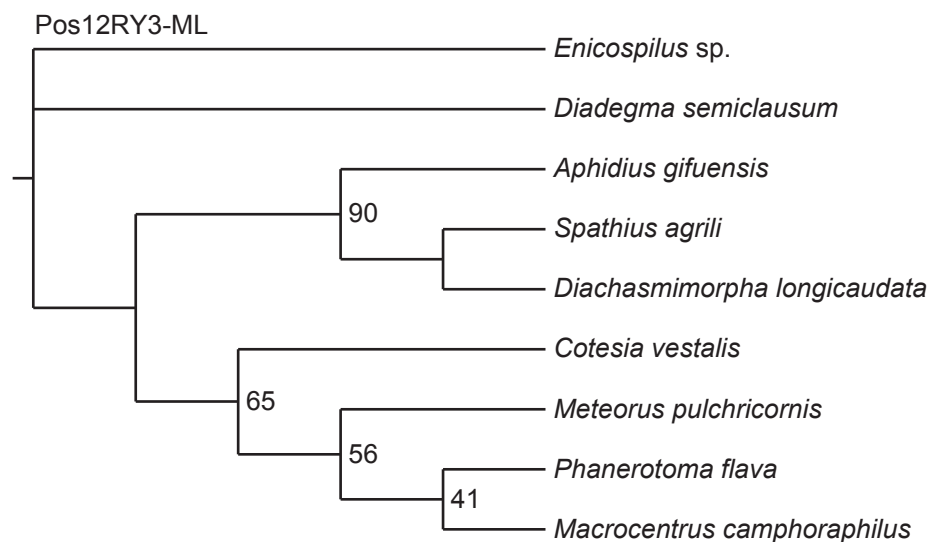

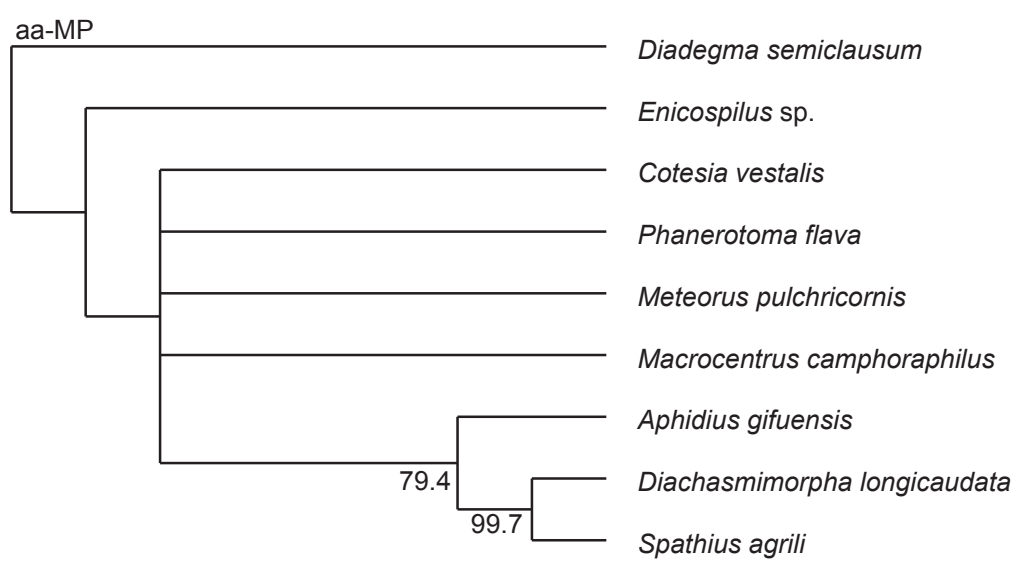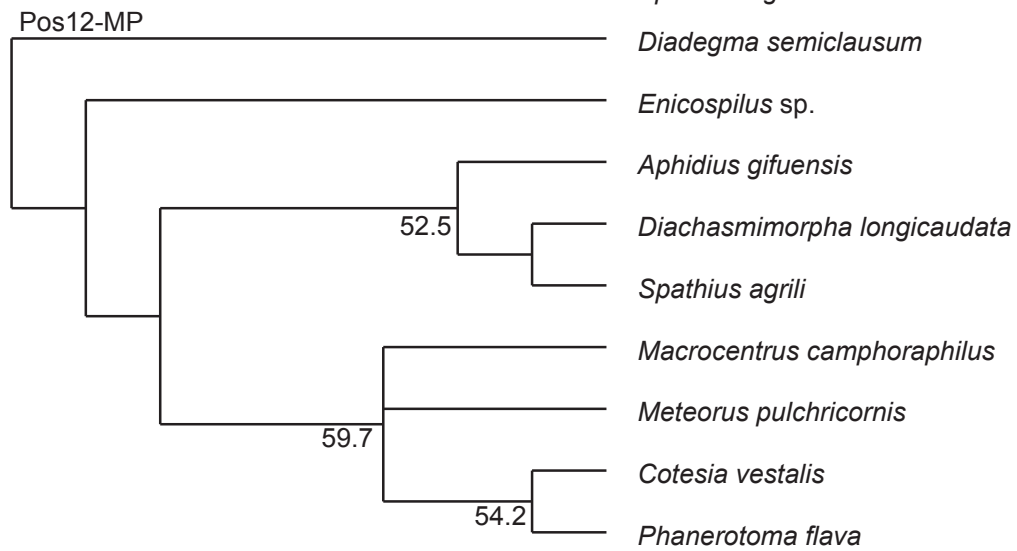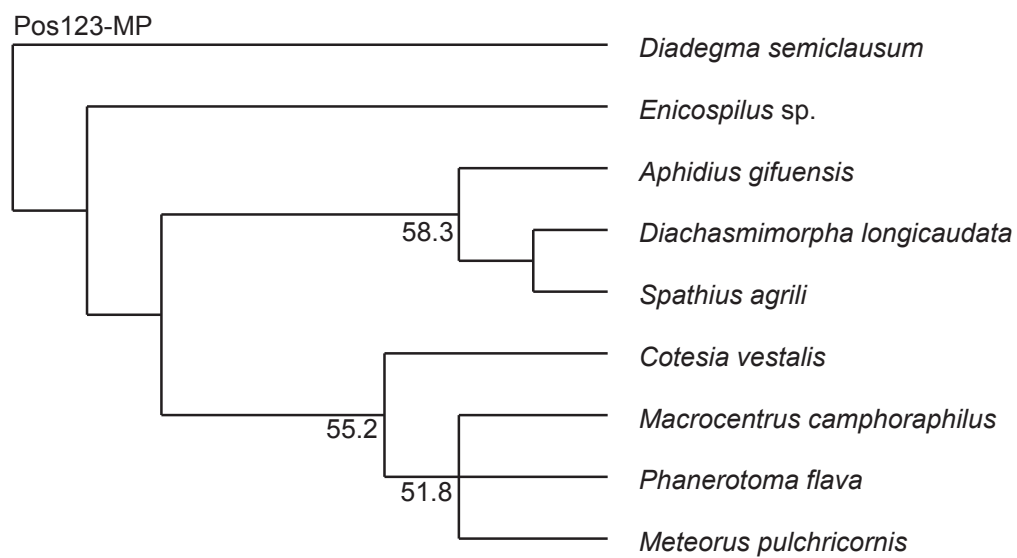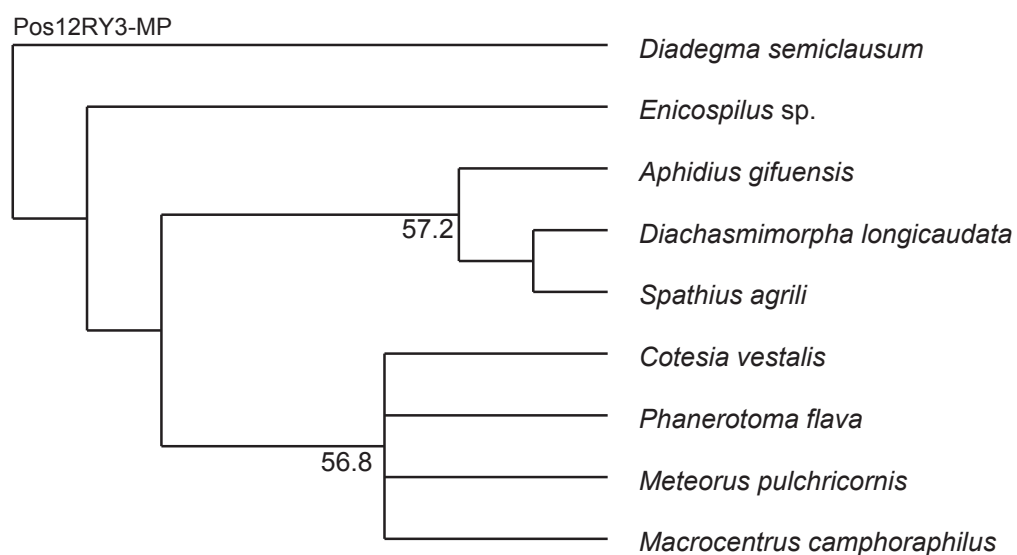

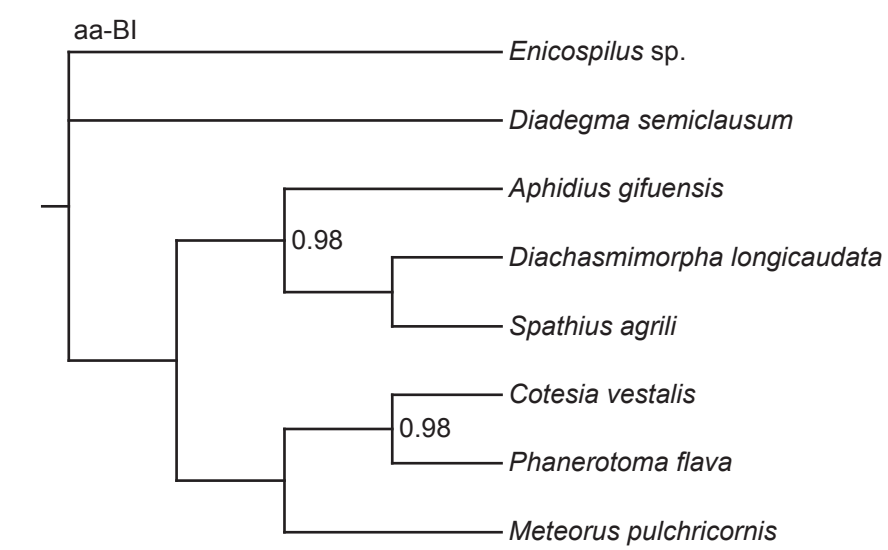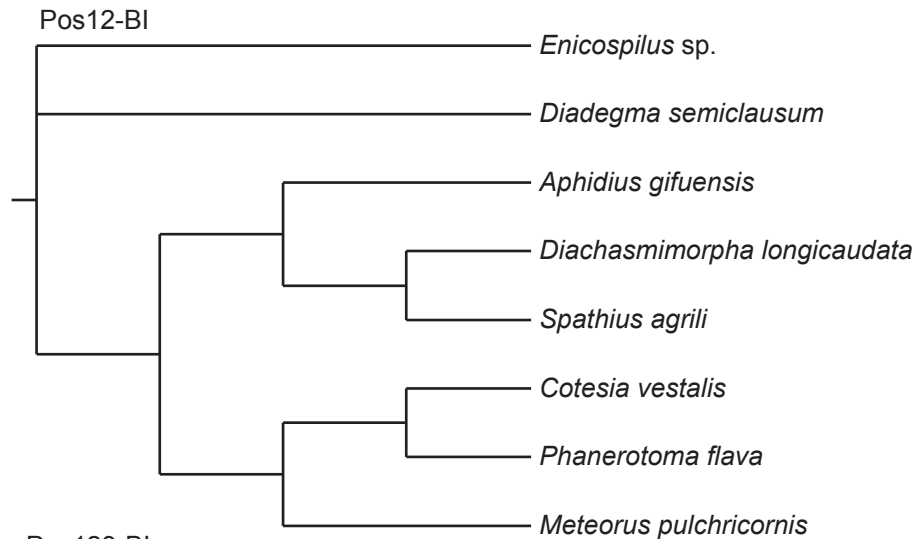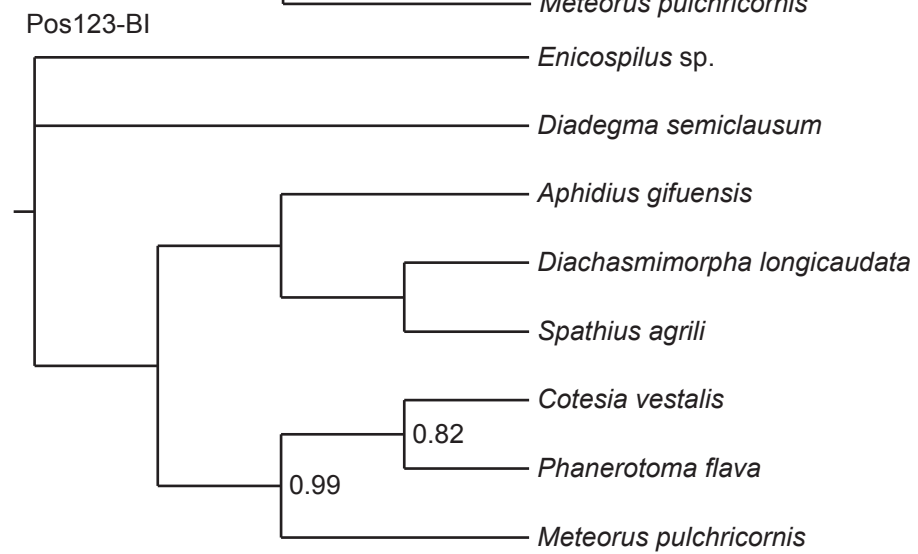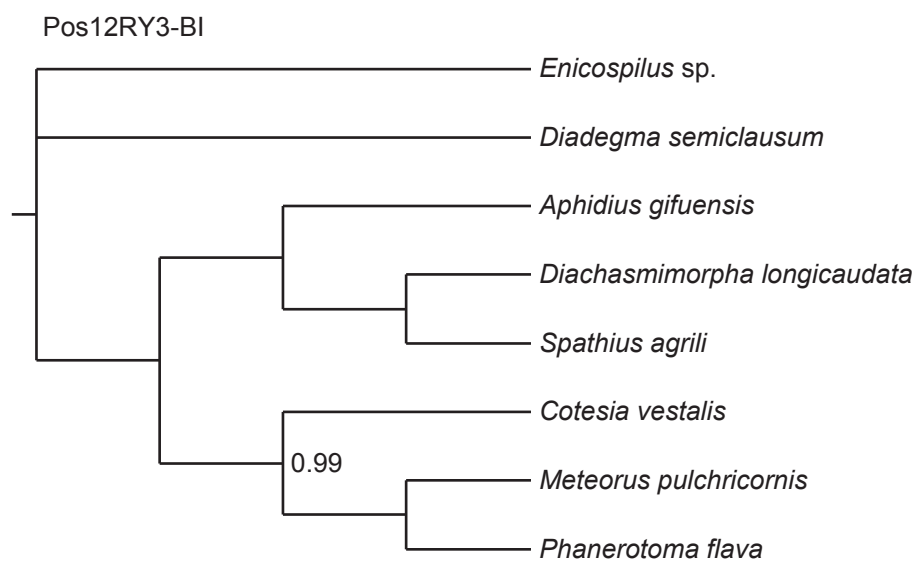

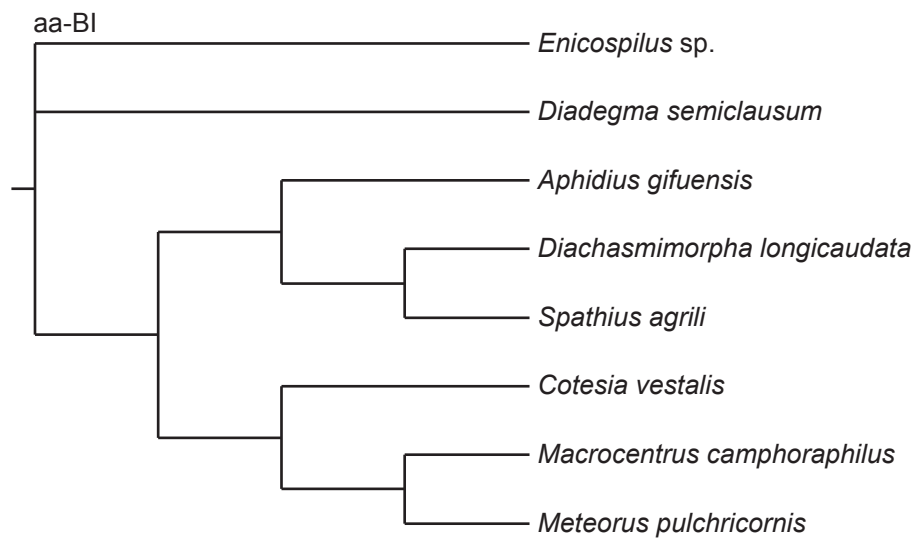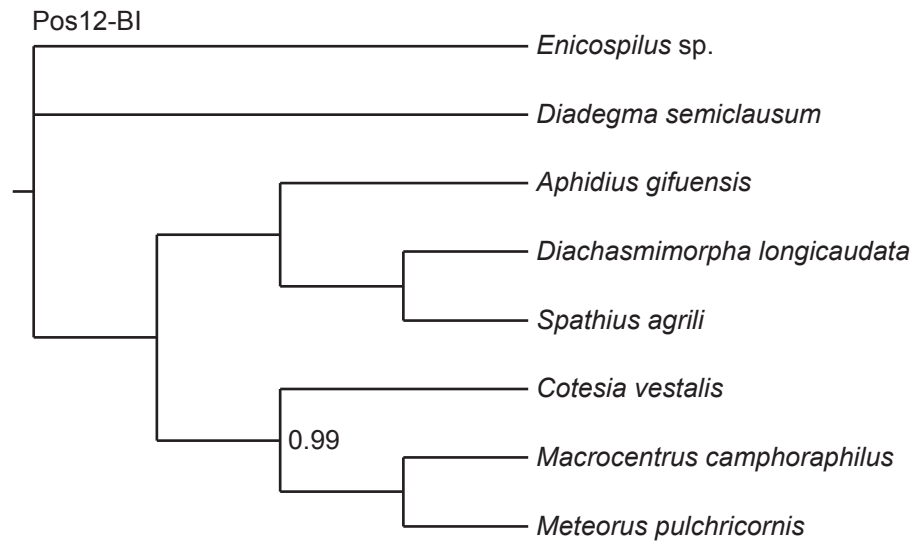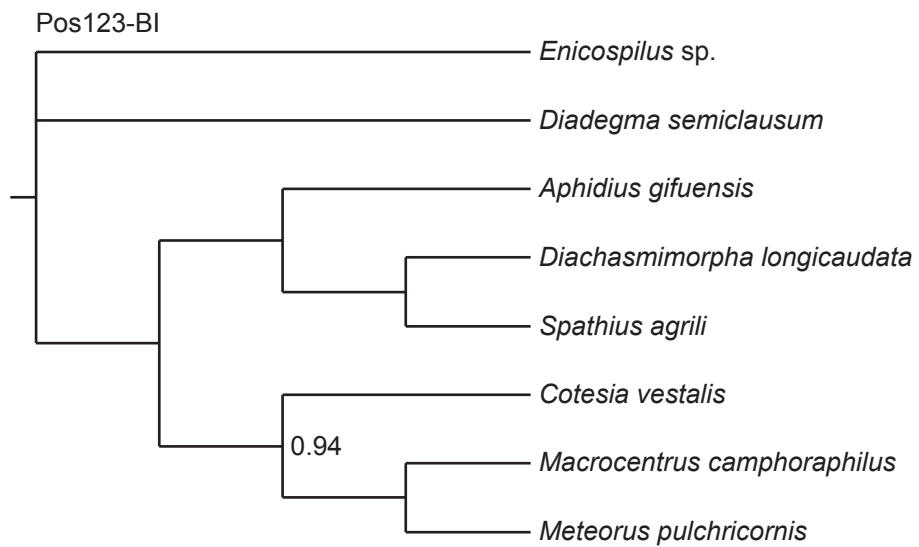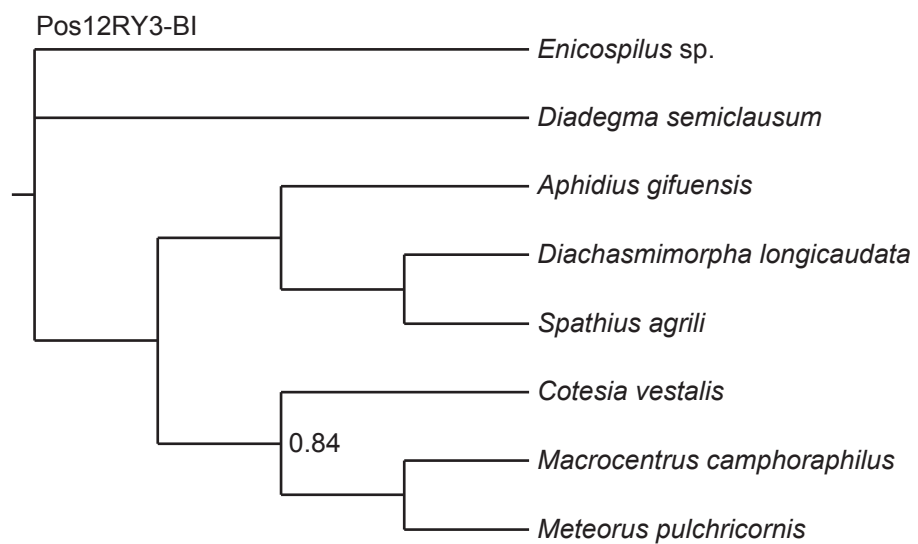

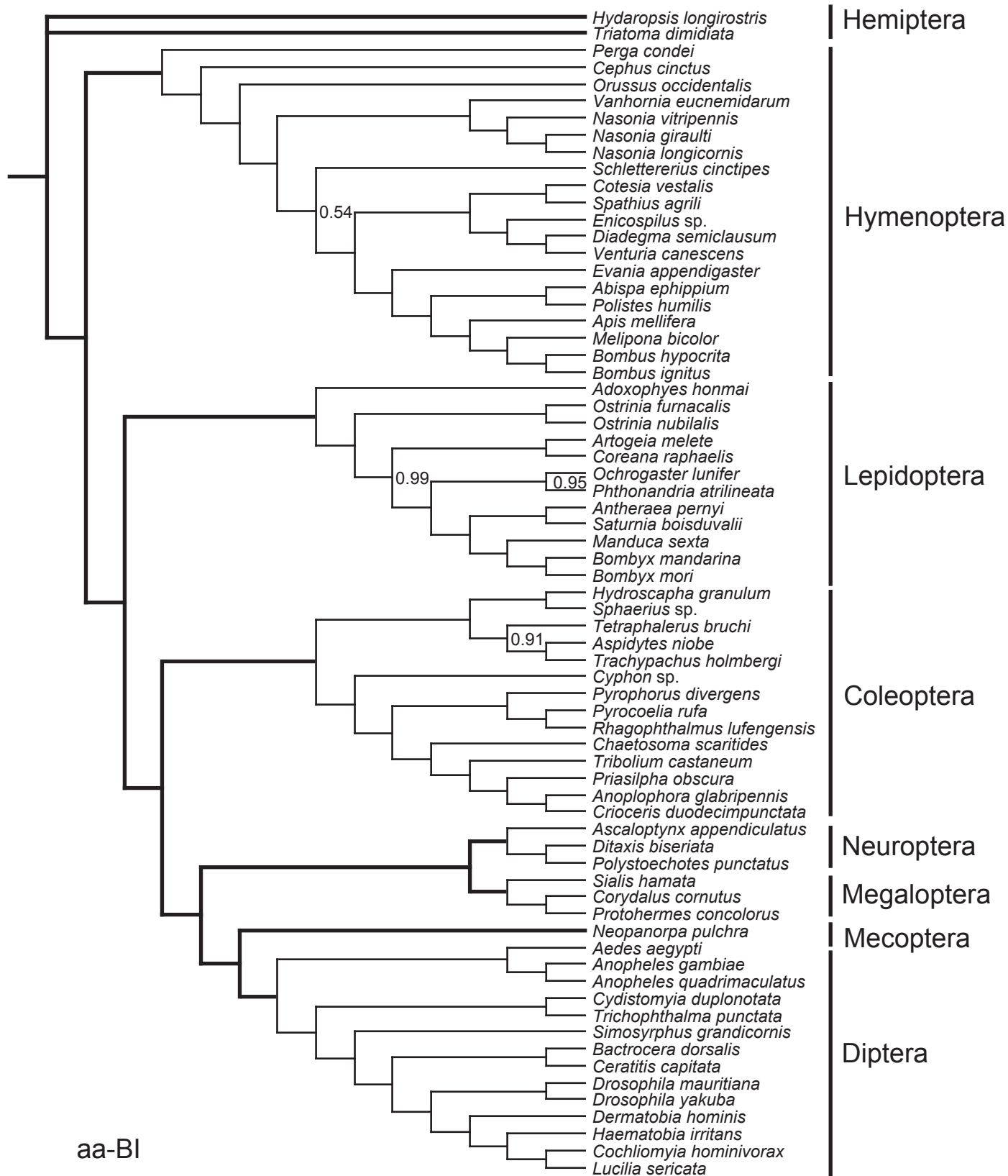

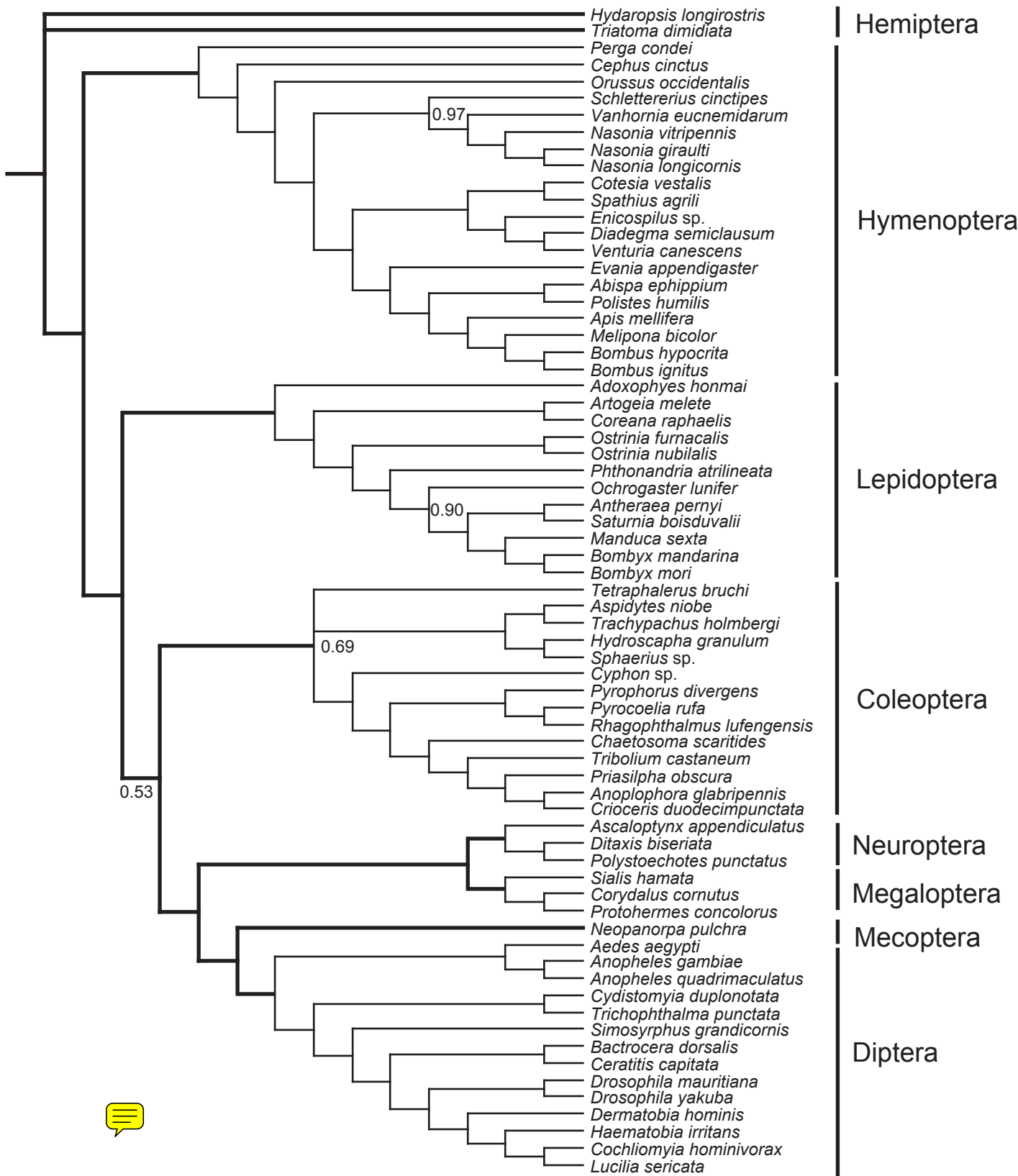

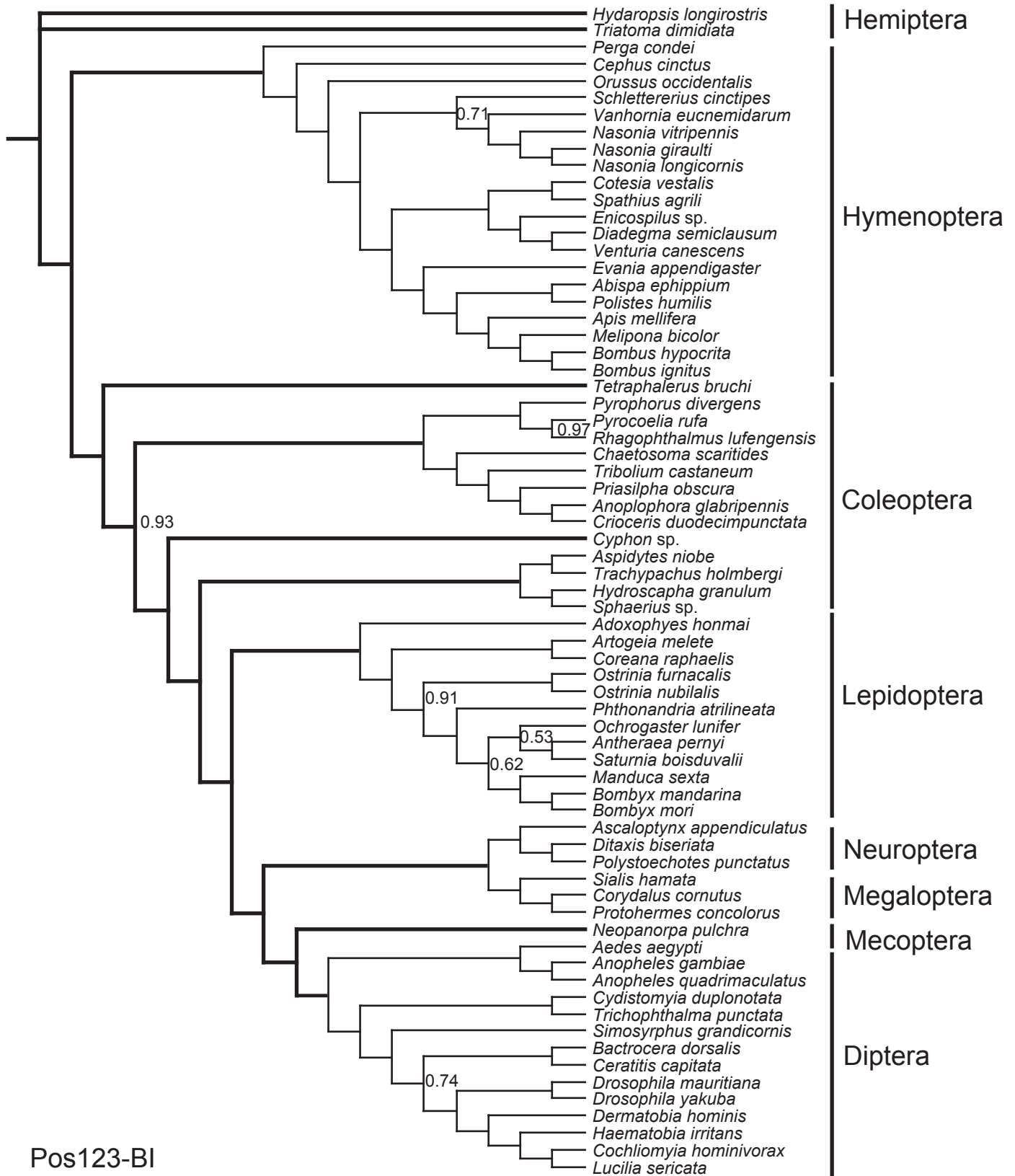

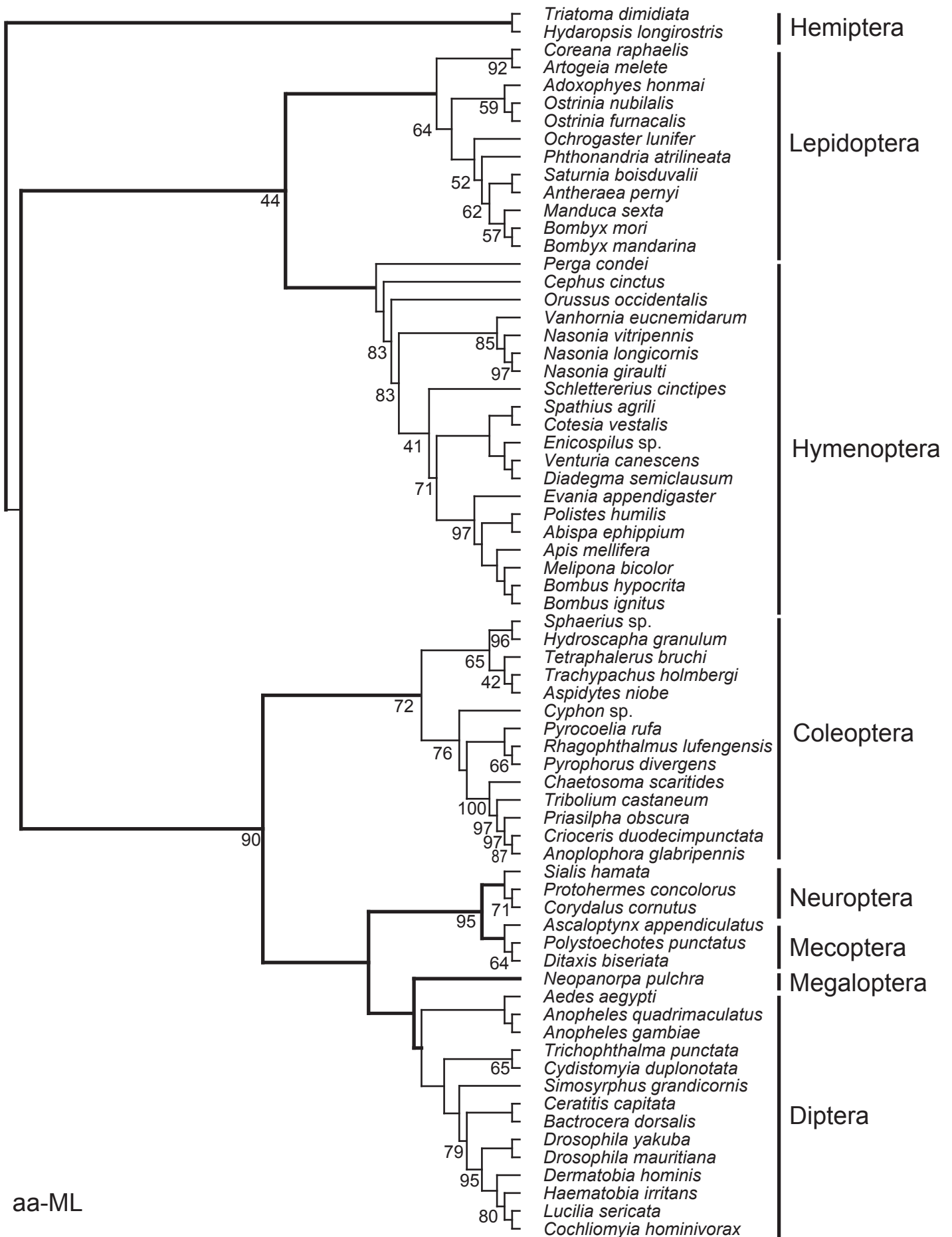

aa-ML

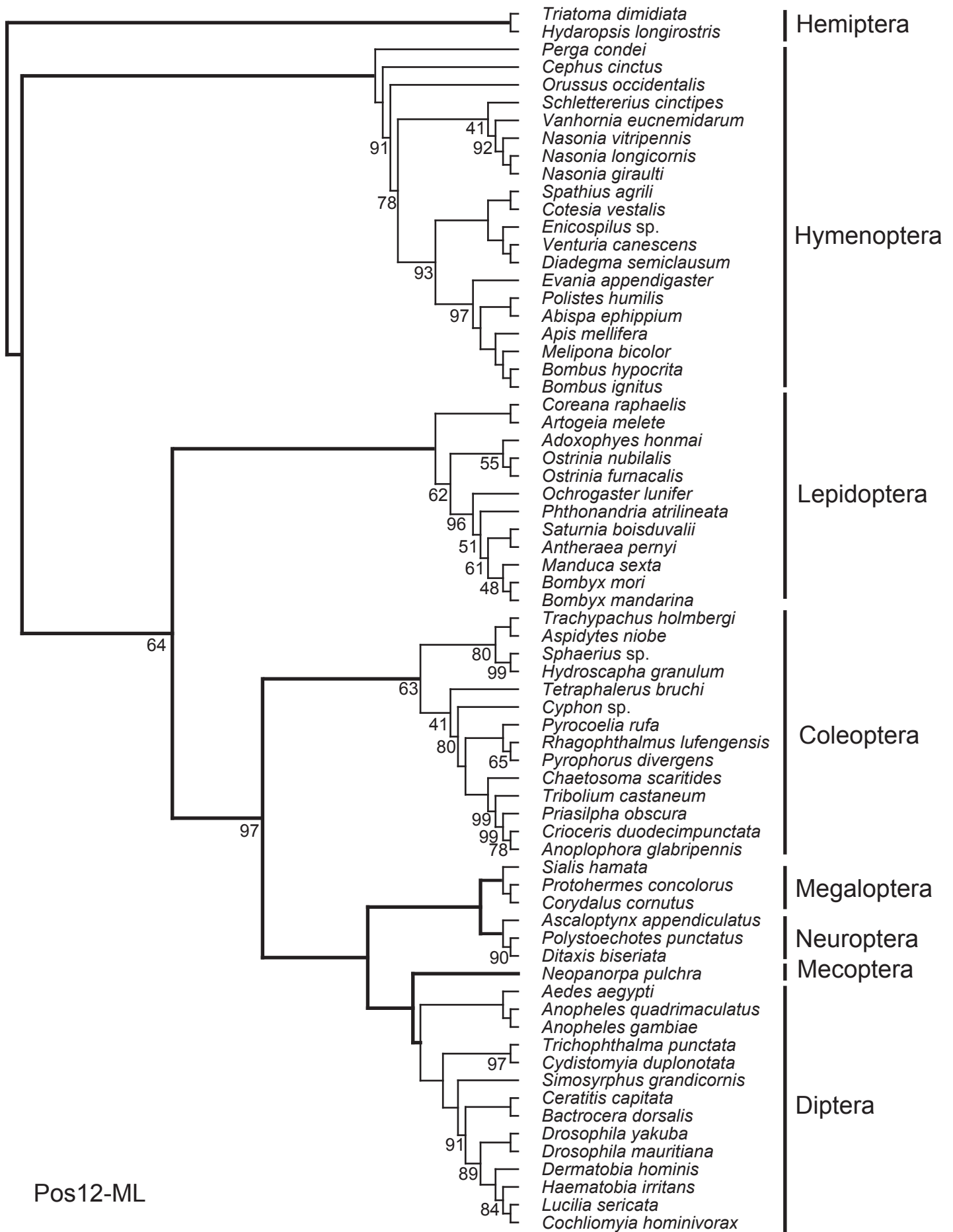

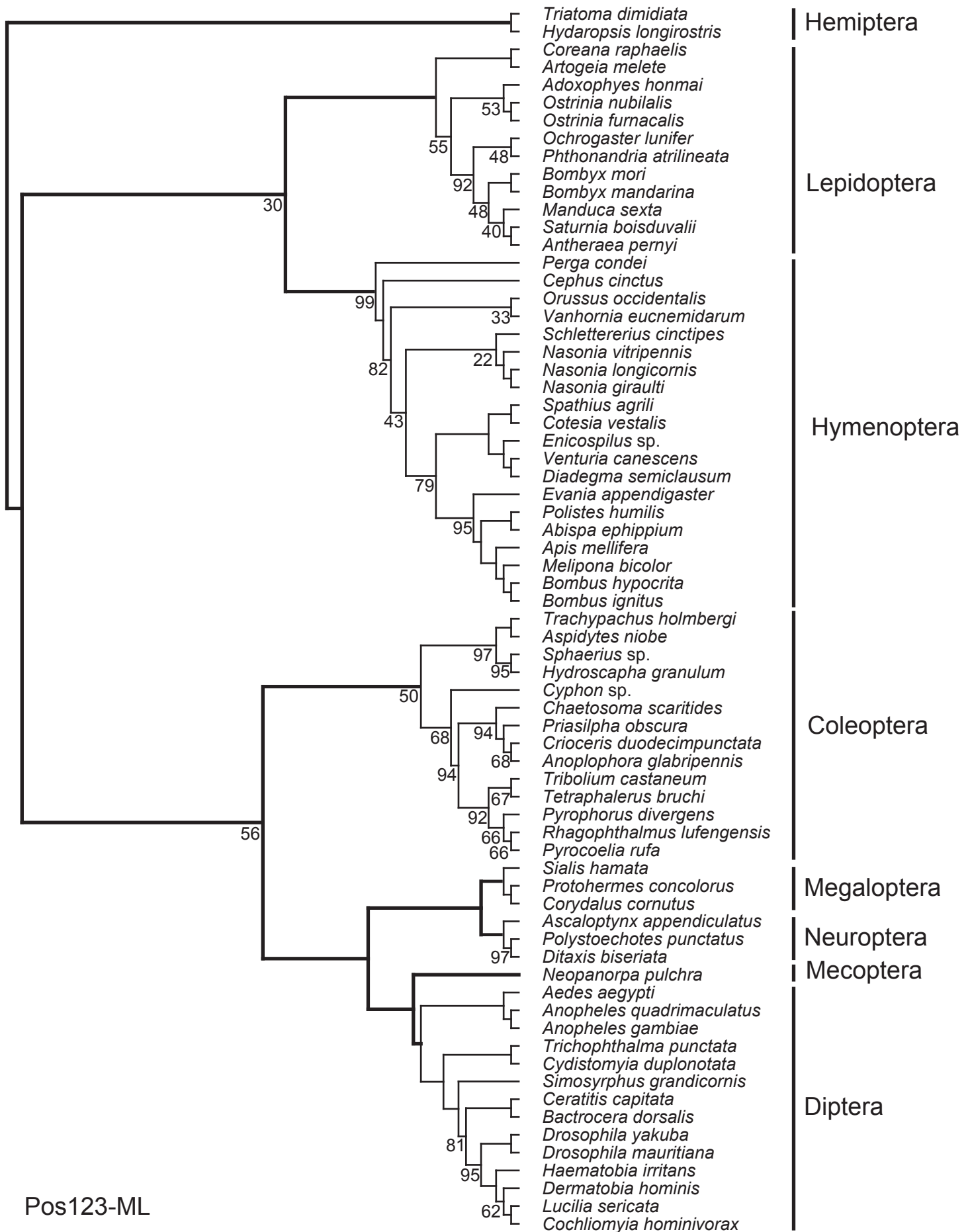

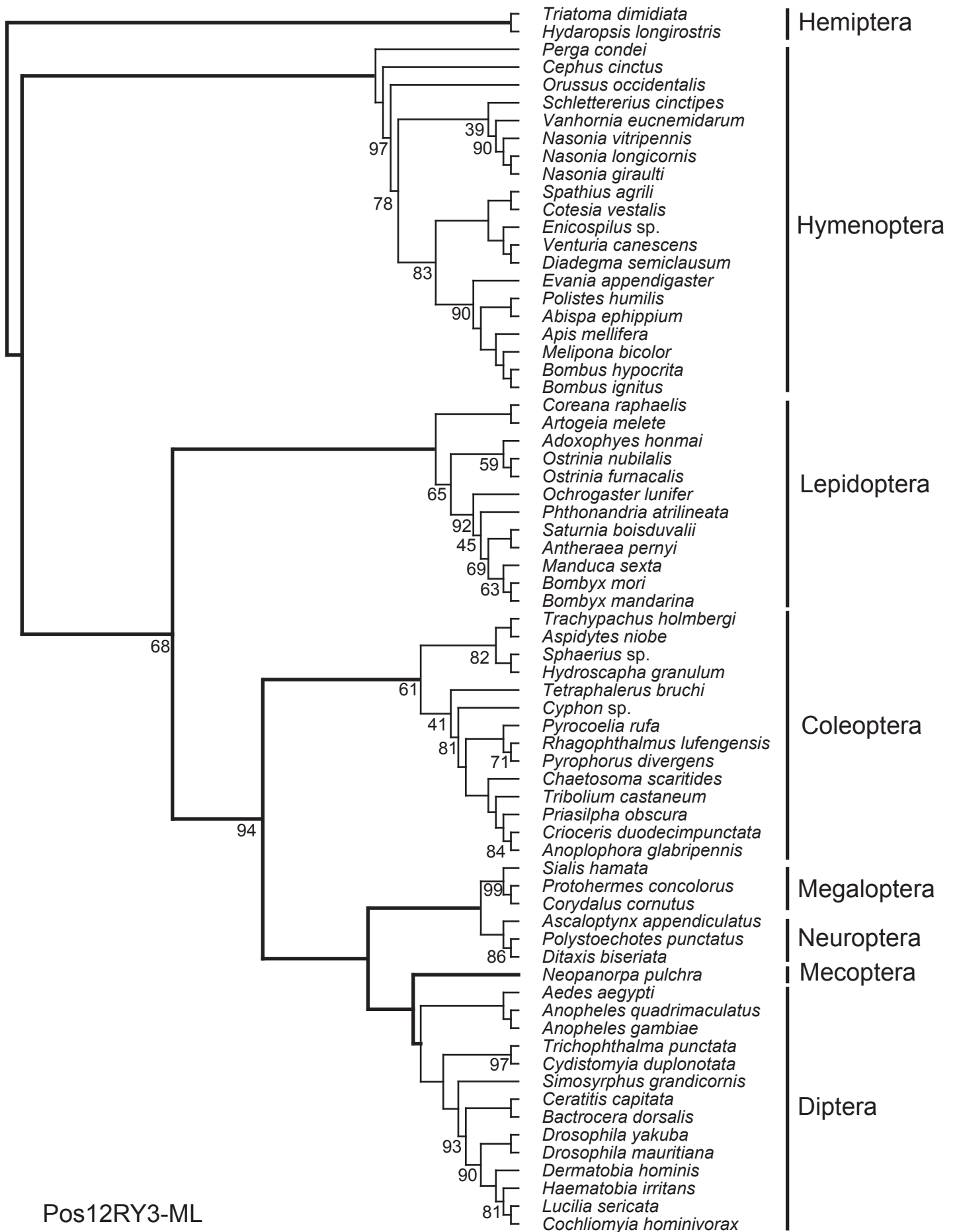

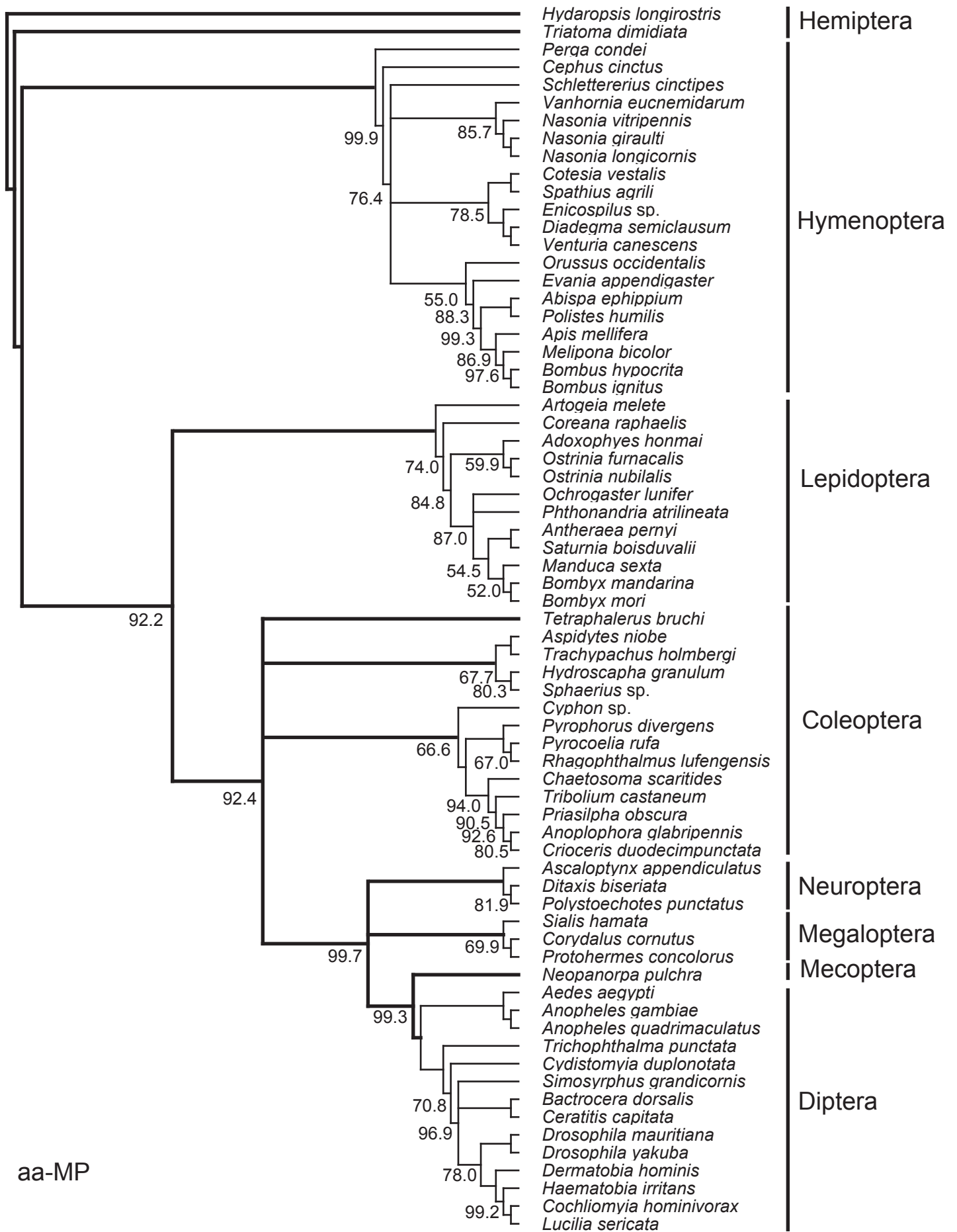

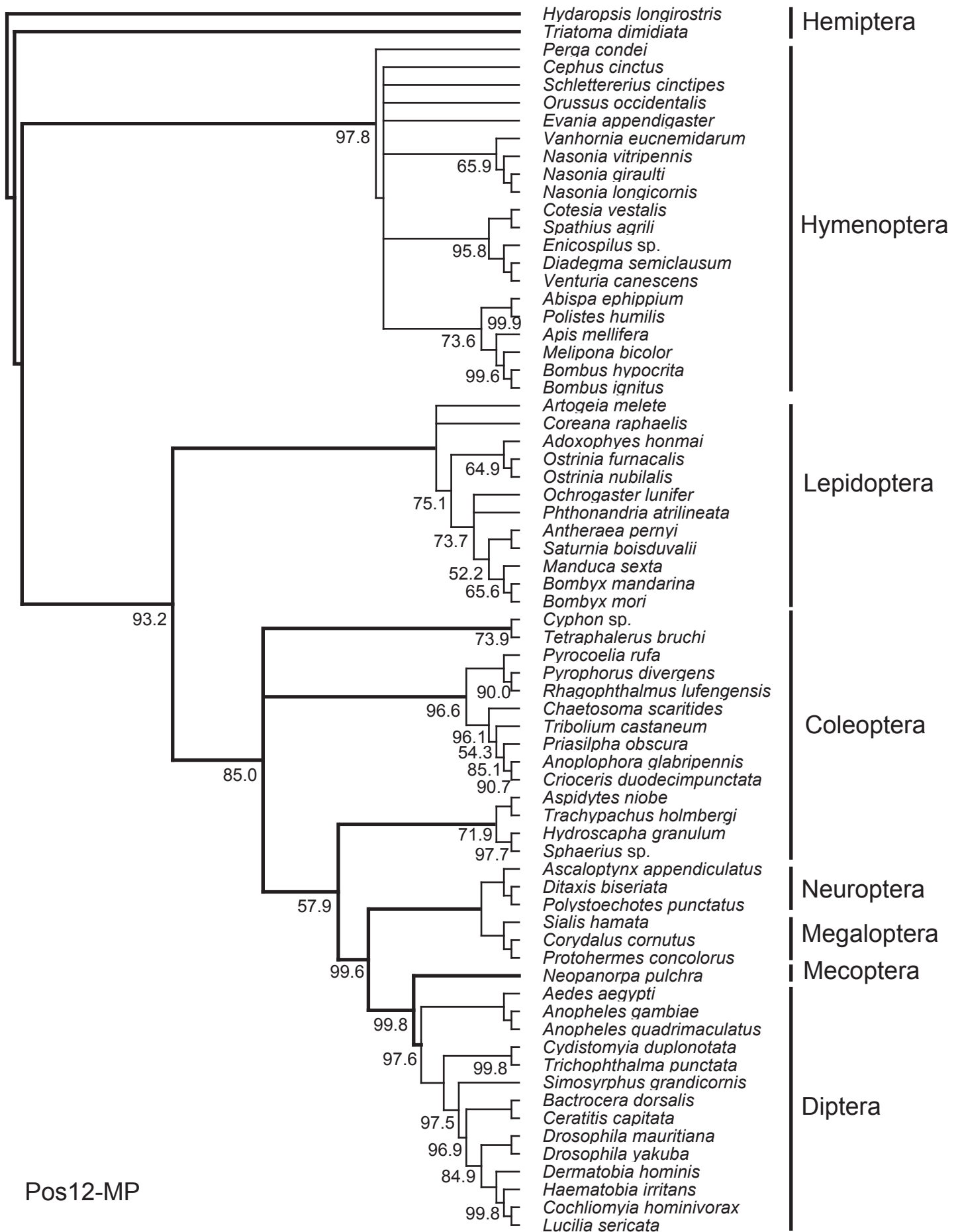

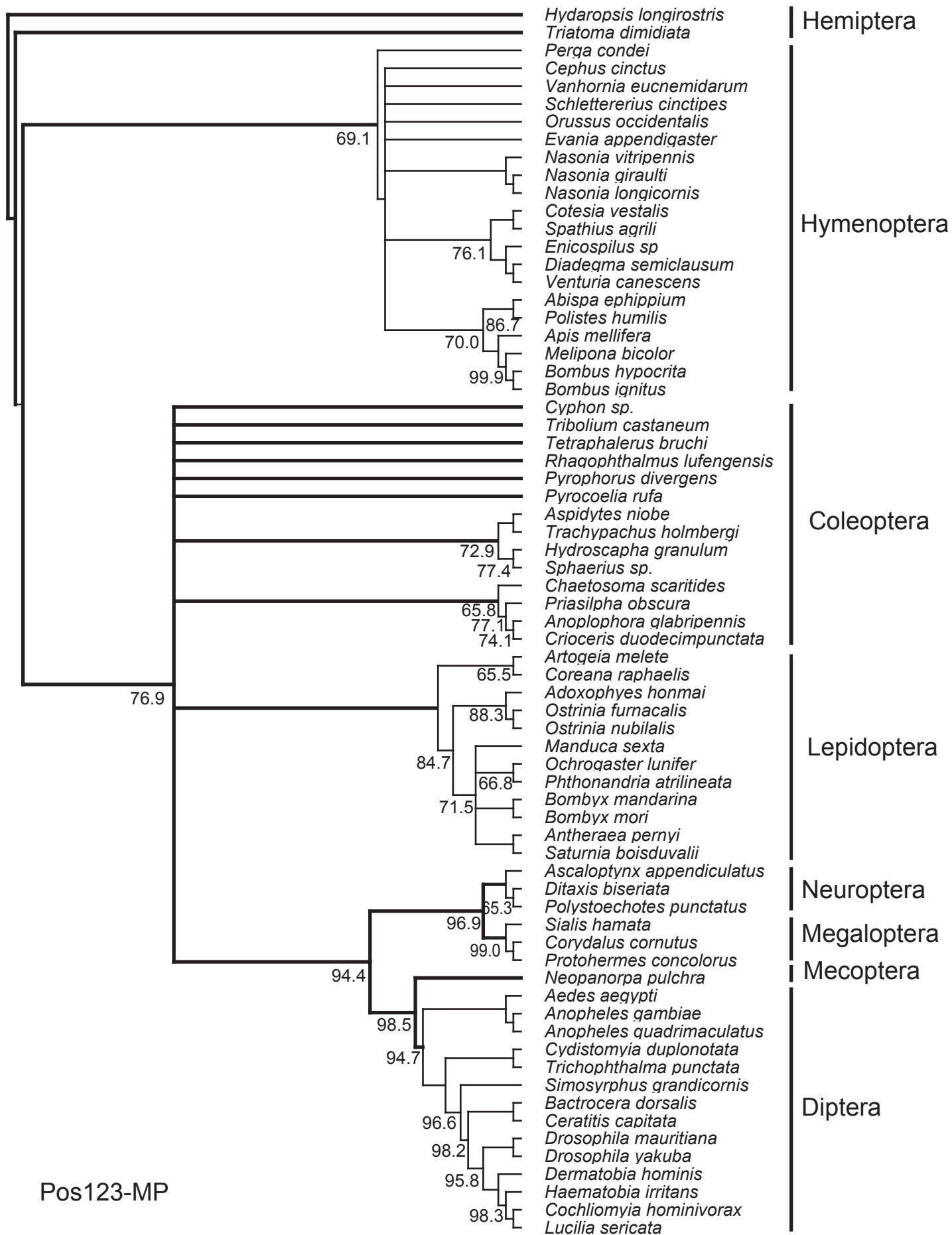

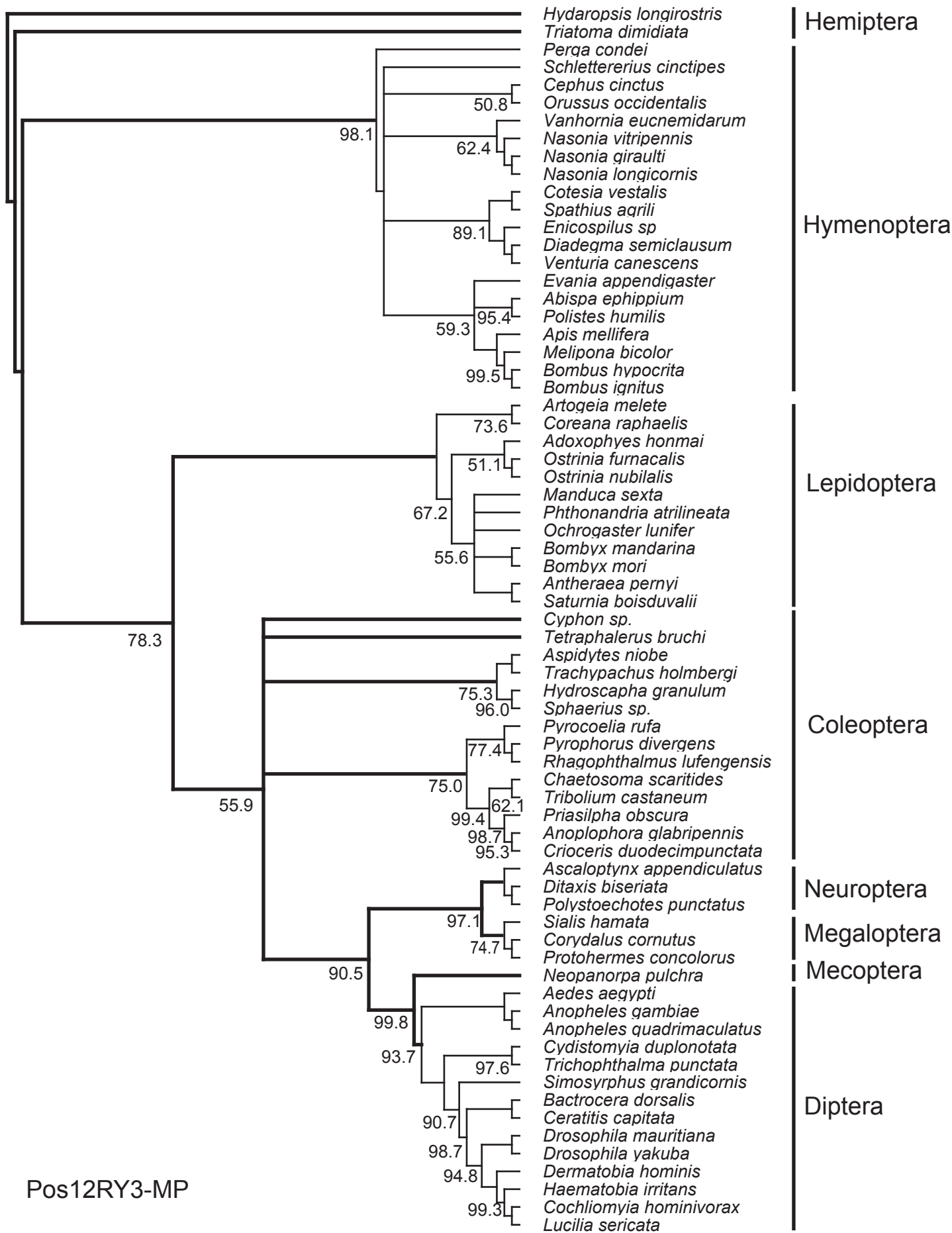

Supplement: Additional file 1 — Phylogenetic trees constructed in this study [file 1471-2164-11-371-S1.PDF]
