# Supplementary material for: Comparative mitogenomics of Braconidae (Insecta: Hymenoptera) and the phylogenetic utility of mitochondrial genomes with special reference to Holometabolous insects
Source: BMC Genomics. 2010 Jun 11;11:371. doi: 10.1186/1471-2164-11-371 (PMC2890569; doi:10.1186/1471-2164-11-371)
Supplement: Additional file 2 — Species used in phylogenetic reconstruction of Holometabola [file 1471-2164-11-371-S2.DOC]

### Additional file 2 – Species used in phylogenetic reconstruction of Holometabola

| Species | Order | Family | Accession number |
| --- | --- | --- | --- |
| *Anoplophora glabripennis* | Coleoptera | Cerambycidae | NC008221 |
| *Crioceris duodecimpunctata* | Coleoptera | Crioceridae | NC003372 |
| *Pyrophorus divergens* | Coleoptera | Elateridae | NC009964 |
| *Pyrocoelia rufa* | Coleoptera | Lampyridae | NC003970 |
| *Chaetosoma scaritides* | Coleoptera | Melyridae | NC011324 |
| *Rhagophthalmus lufengensis* | Coleoptera | Phengodidae | NC010969 |
| *Priasilpha obscura* | Coleoptera | Phloeostichidae | NC011326 |
| *Cyphon* sp. | Coleoptera | Scirtidae | NC011320 |
| *Sphaerius* sp. | Coleoptera | Sphaeriusidae | NC011322 |
| *Tribolium castaneum* | Coleoptera | Tenebrionidae | NC003081 |
| *Trachypachus holmbergi* | Coleoptera | Trachypachidae | NC011329 |
| *Tetraphalerus bruchi* | Coleoptera | Ommatidae | NC011328 |
| *Hydroscapha granulum* | Coleoptera | Hydroscaphidae | NC012144 |
| *Aspidytes niobe* | Coleoptera | Aspidytidae | NC_012139 |
| *Cochliomyia hominivorax* | Diptera | Calliphoridae | NC002660 |
| *Lucilia sericata* | Diptera | Calliphoridae | NC009733 |
| *Anopheles quadrimaculatus* | Diptera | Culicidae | NC000875 |
| *Anopheles gambiae* | Diptera | Culicidae | NC002084 |
| *Aedes aegypti* | Diptera | Culicidae | NC010241 |
| *Dermatobia hominis* | Diptera | Oestridae | NC006378 |
| *Drosophila yakuba* | Diptera | Drosophilidae | NC001322 |
| *Drosophila mauritiana* | Diptera | Drosophilidae | NC005779 |
| *Haematobia irritans irritans* | Diptera | Muscidae | NC007102 |
| *Trichophthalma punctata* | Diptera | Nemestrinidae | NC008755 |
| *Simosyrphus grandicornis* | Diptera | Syrphidae | NC008754 |
| *Cydistomyia duplonotata* | Diptera | Tabanidae | NC008756 |
| *Ceratitis capitata* | Diptera | Tephritidae | NC000857 |
| *Bactrocera dorsalis* | Diptera | Tephritidae | NC008748 |
| *Triatoma dimidiata* | Hemiptera | Reduviidae | NC002609 |
| *Hydaropsis longirostris* | Hemiptera | Coreidae | EU427337 |
| *Apis mellifera ligustica* | Hymenoptera | Apidae | NC001566 |
| *Melipona bicolor* | Hymenoptera | Apidae | NC004529 |
| *Bombus ignitus* | Hymenoptera | Apidae | NC010967 |
| *Abispa ephippium* | Hymenoptera | Eumenidae | NC011520 |
| *Diadegma semiclausum* | Hymenoptera | Ichneumonidae | EU871947 |
| *Spathius agrili* | Hymenoptera | Braconidae | FJ387020 |
| *Cotesia plutellae* | Hymenoptera | Braconidae | FJ154897 |
| *Perga condei* | Hymenoptera | Pergidae | AY787816 |
| *Nasonia vitripennis* | Hymenoptera | Pteromalidae | EU746615, EU746613, EU746610 |
| *Nasonia longicornis* | Hymenoptera | Pteromalidae | EU746612, EU746616 |
| *Nasonia giraulti* | Hymenoptera | Pteromalidae | EU746609, EU746611, EU746614 |
| *Vanhornia eucnemidarum* | Hymenoptera | Vanhoridae | NC008323 |
| *Polistes humilis* | Hymenoptera | Vespidae | EU024653 |
| *Evania appendigaster* | Hymenoptera | Evanidae | FJ593187 |
| *Bombus hypocrita sapporoensis* | Hymenoptera | Pteromalidae | NC011923 |
| *Enicospilus sp* | Hymenoptera | Pteromalidae | FJ478177 |
| *Cephus cinctus* | Hymenoptera | Pteromalidae | FJ478173 |
| *Orussus occidentalis* | Hymenoptera | Stephanidae | FJ478174 |
| *Schlettererius cinctipes* | Hymenoptera | Vanhoridae | FJ478175 |
| *Venturia canescens* | Hymenoptera | Vespidae | FJ478176 |
| *Bombyx mori* | Lepidoptera | Bombycidae | NC002355 |
| *Bombyx mandarina* | Lepidoptera | Bombycidae | NC003395 |
| *Phthonandria atrilineata* | Lepidoptera | Geometridae | NC010522 |
| *Coreana raphaelis* | Lepidoptera | Lycaenidae | NC007976 |
| *Adoxophyes honmai* | Lepidoptera | Tortricidae | NC008141 |
| *Ochrogaster lunifer* | Lepidoptera | Notodontidae | NC011128 |
| *Artogeia melete* | Lepidoptera | Pieridae | NC010568 |
| *Ostrinia nubilalis* | Lepidoptera | Pyralidae | NC003367 |
| *Ostrinia furnacalis* | Lepidoptera | Pyralidae | NC003368 |
| *Antheraea pernyi* | Lepidoptera | Saturniidae | NC004622 |
| *Saturnia boisduvalii* | Lepidoptera | Saturniidae | NC010613 |
| *Manduca sexta* | Lepidoptera | Sphingidae | NC010266 |
| *Neopanorpa pulchra* | Mecoptera | Panorpidae | NC013180 |
| *Corydalus cornutus* | Megaloptera | Corydalinae | NC011276 |
| *Protohermes concolorus* | Megaloptera | Corydalidae | NC011524 |
| *Sialis hamata* | Megaloptera | Sialidae | NC013256 |
| *Polystoechotes punctatus* | Neuroptera | Polystoechotidae | NC011278 |
| *Ascaloptynx appendiculatus* | Neuroptera | Ascalaphidae | NC011277 |
| *Ditaxis biseriata* | Neuroptera | Mantispidae | NC013257 |
